# Extending the applicability of the Goldschmidt tolerance factor to arbitrary ionic compounds

**DOI:** 10.1038/srep23592

**Published:** 2016-04-01

**Authors:** Toyoto Sato, Shigeyuki Takagi, Stefano Deledda, Bjørn C. Hauback, Shin-ichi Orimo

**Affiliations:** 1 Institute for Materials Research, Tohoku University, Sendai 980-8577, Japan; 2 Institute for Energy Technology, Physics Department, Kjeller, NO-2027, Norway; 3 WPI-Advanced Institute for Materials Research, Tohoku University, Sendai 980-8577, Japan

## Abstract

Crystal structure determination is essential for characterizing materials and their properties, and can be facilitated by various tools and indicators. For instance, the Goldschmidt tolerance factor (*T*) for perovskite compounds is acknowledged for evaluating crystal structures in terms of the ionic packing. However, its applicability is limited to perovskite compounds. Here, we report on extending the applicability of *T* to ionic compounds with arbitrary ionic arrangements and compositions. By focussing on the occupancy of constituent spherical ions in the crystal structure, we define the ionic filling fraction (IFF), which is obtained from the volumes of crystal structure and constituent ions. Ionic compounds, including perovskites, are arranged linearly by the IFF, providing consistent results with *T*. The linearity guides towards finding suitable unit cell and composition, thus tackling the main obstacle for determining new crystal structures. We demonstrate the utility of the IFF by solving the structure of three hydrides with new crystal structures.

Every compound can be identified by a unique arrangement of the atoms in the crystal structure, which determines the inter–atomic distances and the compositional ratios and thus helps understanding specific material properties. For this reason, crystal structure determination and analysis are essential not only from a mere crystallographic viewpoint, but also for numerous fields of materials research, chemistry, physics and life sciences.

The evaluation of crystal structures can be facilitated by various tools and indicators. For instance, Goldschmidt tolerance factor (*T* ) of perovskite compounds ABX_3_ (A and B: metal cations, X: chalcogen or halogen group) is widely used to assess the geometric stability and distortion of crystal structures in terms of the constituent ionic packing. The *T* is defined by ratios of constituent ionic radii of A, B and X as *T* = (*R*_A_ + *R*_X_)/√2(*R*_B_ + *R*_X_), where *R*_A_, *R*_B_ and *R*_X_ are the ionic radii of A, B and X, respectively (c.f. Fig. 1a). The ideal perovskite compounds adopt a cubic close packed structure with *T* = 1. When the ratio of the ionic radii deviates from the ideal value (*T *≠ 1), a geometric strain and crystal distortions arise. As the deviation from *T* = 1 becomes greater, the crystal adopts structures of lower symmetry than the cubic one. Thus by calculating *T*, the crystalline structure can be predicted and its geometric strain and stability evaluated. At the same time, the *T* can be used to estimate the compatibility of different ions with a crystalline structure. Because of its simplicity and practicality, the *T* is used extensively in a wide variety of fundamental and applied studies^1^,^2^,^3^,^4^,^5^.

The application of the *T* has been recently extended to ABX_3_ hydrides where hydrogen is the anion X^6^,^7^. However, it is difficult to apply it to other hydrides that do not possess a perovskite-type crystal structure. Due to the high reactivity of hydrogen, the majority of hydrides occur in an extensive variety of chemically and structurally diverse compounds^7^,^8^,^9^,^10^,^11^,^12^,^13^,^14^,^15^,^16^,^17^,^18^,^19^. Such diversity is complicated by the experimental challenges which limit our ability to determine crystal structures and compositional ratios. This can be a major drawback during the initial steps of the crystal structure determination process, when possible candidates for structural models are identified^20^,^21^. In this respect, it would be useful to extend the applicability of the *T*, which could provide information on the geometrical stability of crystalline structures and not be restricted to ABX_3_ perovskite structures.

In this communication, we propose to extend the applicability of the *T* to be used also for ionic compounds with arbitrary ionic arrangements and compositions including hydrides. In order to extract ionic packing information from arbitrary ionic compounds as with the concept of the *T*, those compounds can be characterized by two criteria: the volume of the unit cell (*V*_unit_) normalized by number of formula units (*Z*) and the total volume of constituent ions that reflects the composition ratio (*V*_ion_). Although those quantities, *V*_unit_ and *V*_ion_, have been previously used for crystal structure determinations of hydrides^7^,^16^,^17^,^18^,^19^, we expanded the utility of the *V*_unit_ and the *V*_ion_ into various ionic compounds with hydrides, oxides and halides focusing on the relation between the *V*_unit_ normalized by *Z*, (*V*_unit_/*Z*), and the *V*_ion_. The relation between the *V*_unit_/*Z* and the *V*_ion_ can be described as occupancy of constituent spherical ion in the crystal structure. We refer to it as the ionic filling fraction (IFF). The IFF surprisingly leads to well organize crystal structures of arbitrary ionic compounds. Here, we use the IFF for assessment of ionic packing of crystal structures from a geometric point of view and discuss how this can be related to the *T*. In addition, we demonstrate the suitability of the IFF for determining the crystal structure of three Al–based complex hydrides (so–called alanates) with new crystal structures, LiCa(AlH_4_)_3_, LiCaAlH_6_ and Sr(AlH_4_)Cl, in which both mixed cations or anions coexist. Such compounds are attractive for fundamental understanding of crystal structures for related complex hydrides which are interests for high gravimetric hydrogen applications. It is thus important to understand their structural features for further progress^9^,^10^,^11^,^12^,^13^. Our results show that the IFF is able to comprehensively classify the crystal structures of various ionic compounds including perovskite compounds and proving a new indicator for determining crystal structures.

## Results

### Ionic filling fraction (IFF) for ionic compounds

**Figure 1:**
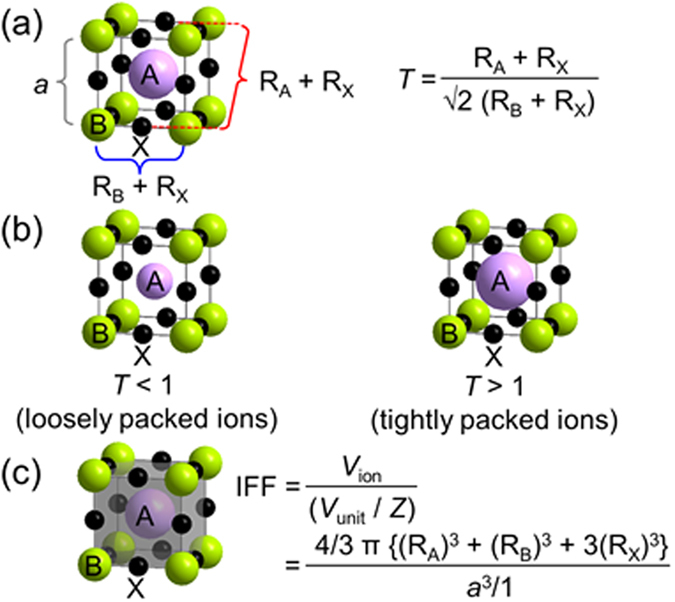
Ionic packing in an ideal cubic perovskite structure. (**a**) concept of *T*, (**b**) loosely packed crystal structure with a small radius of A with *T* < 1 and tightly packed crystal structure with a large radius of A with *T* > 1, and (**c**) concept of the IFF in an ideal cubic perovskite structure with a lattice constant *a* and *Z* = 1 (purple sphere: A; lime spheres: B; black spheres: X; Grey cuboid: *V*_unit_/*Z*; purple + lime + 3 × black spheres: *V*_ion_). The IFF shows spherical ionic volume fraction in the crystal structure (grey cuboid).

We firstly consider the Goldschmidt tolerance factor (*T*). In an ideal cubic perovskite structure with a lattice constant *a, T* is defined by the ratio of three kinds of ionic radii as shown Fig. 1a. Assuming fixed values of radii of B and X for a given lattice constant *a, T* < 1 corresponds to a loosely packed crystal structure with small radius of A and *T *> 1 corresponds to a tightly packed crystal structure with large radius of A (Fig. 1b). This indicates that *T* can also be represented by the occupancy of constituent spherical ions in the crystal structure. Expanding the concept in terms of the occupancy of constituent spherical ions in the crystal structure, allows extending the applicability of *T* to various kinds of ionic compounds (details are described in the next section).

In order to extract occupancy of constituent spherical ions in the crystal structure from different crystal structures of various kinds of ionic compounds, we need to secondly define a standard approach for those ionic compounds. The repeating unit of a crystalline compound is determined by the unit cell given by the lattice constants (*a, b, c, α, β* and *γ*). However, it might be more convenient to consider the relative sizes of the asymmetric unit in the crystal by evaluating the volume of the unit cell (*V*_unit_) normalized by the number of formula units (*Z*): *V*_unit_/*Z*. On the other hand, crystal structures are also dependent on the constituent components and their relative fraction (composition ratio). To observe this dependence, composition ratios can be represented in terms of volume using the same units as those for *V*_unit_/*Z.* Assuming spherical ions with a volume governed by their Shannon radii^22^; the total volume occupied by the ions (*V*_ion_) can be calculated according to the stoichiometric ratio of the ionic compounds. Then, the ratio between *V*_unit_/*Z* and *V*_ion_ represents the occupancy of constituent spherical ion in the crystal structure for ionic compounds with arbitrary ionic arrangements and composition. We will refer to the concept, which is the ratio between *V*_unit_/*Z* and *V*_ion_, as the ionic filling fraction (IFF). The schematic figure for the IFF to an ideal cubic perovskite compound shows in Fig. 1c. Although the *T* is limited in fixed number of constituent ions with perovskite–type structure, it should be noted that the IFF concept can be flexibly responded any modifications of crystal structures and numbers of constituent ions.

**Figure 2:**
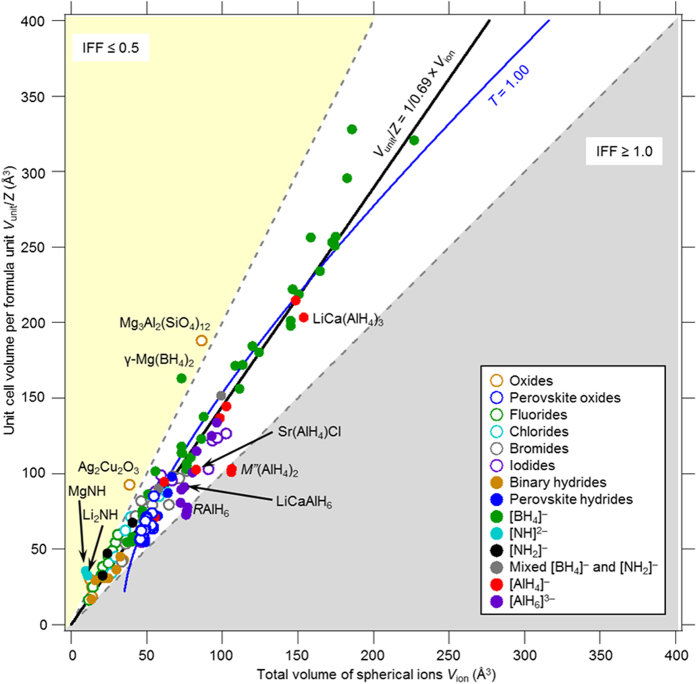
IFF plot for ionic compounds with arbitrary ionic arrangements and compositions. IFFs for 137 ionic compounds are shown (Black line: *V*_unit_/*Z* = 1/0.69 × *V*_ion_; blue curve: *T* = 1.0; yellow area: IFF ≤ 0.5; grey area: IFF ≥ 1.0). The inverse of the slope on *V*_unit_/*Z* = 1/0.69 × *V*_ion_ corresponds to the IFF (=0.69).

Using the IFF, we extend the applicability to ionic compounds including hydrides with a variety of chemically and structurally diverse compounds. 1.40 Å is used as the radius of the H^−^ ion^6^. In the hydrides, elements belonging to Group 6–15 in the periodic table are known to primarily form complex anion with hydrogen^8^,^9^,^10^,^13^,^15^,^16^,^17^,^18^,^19^. The complex anions ionically bond with metal cations in the formation of complex hydrides. In case of complex anions formed with multiple elements, the thermochemical radius is used. It is estimated from the Glasser generalization of Kapustinskii’s equation for lattice energy of ionic compounds, is used (The definition of the thermochemical radius and coordination numbers (CNs) is presented in the Supplementary information)^23^,^24^,^25^,^26^. This enables the estimation of the radius of a complex anion, which is assumed as a rigid spherical ion. Figure 2 shows a plot of *V*_unit_/*Z* vs. *V*_ion_ for typical oxides, halides and hydrides (a total of 137 compounds). The *V*_unit_/*Z* and *V*_ion_ values of these compounds are listed in Supplementary Tables 1–14. The inverse of the slope of straight lines that pass through the origin in each plot represents the IFF of spherical ions in the crystal structures. We will refer to this plot as the IFF plot. Although IFF is dependent on the crystal structure of each ionic compound (for the closest packing crystal structure based on a single component, IFF = 0.74), it should be noted that the data points are mostly distributed around the best linear fit at *V*_unit_/*Z* = 1/0.69 × *V*_ion_, corresponding to IFF of 0.69. This suggests that ionic compounds adopt a constant IFF value (0.69). The constant IFF is close to the IFF of a body–centred cubic crystal structure containing a single component (IFF = 0.68). The hydrides in Fig. 2 are ionic hydrides and have stoichiometric compositions to fulfil electro–neutrality. In case of non–stoichiometric hydrides, hydrogen is dissolved in the metal lattice and located in interstitial sites of the metal lattice^15^. For such non–stoichiometric hydrides it is difficult to define the size of hydrogen. If the size of hydrogen in the interstitial sites could be precisely defined, it could be possible to include non–stoichiometric hydrides in the plot of Fig. 2.

In the IFF plots, the region for which *V*_unit_/*Z* ≧ 1/0.69 × *V*_ion_ denotes crystal structures containing voids. In particular, ionic compounds with IFF ≦0.50 (*V*_unit_/*Z* ≧ 1/0.50 × *V*_ion_, yellow region in Fig. 2) are characterized by porous crystal structures^10^,^27^,^28^,^29^,^30^,^31^. On the other hand, ionic compounds for which *V*_unit_/*Z *≦ 1/0.69 × *V*_ion_ are characterized by tightly packed arrangements of ions resulting in dense crystal structures. It is worth noting that some compounds*, M*″(AlH_4_)_2_ (*M*″: Sr, Eu)^32^ and *R*AlH_6_ (*R*: La, Ce, Pr, Nd)^33^ lie in the area with IFF ≧ 1.0 (grey region) which implies *V*_unit_/*Z *≦ *V*_ion_, This region should feature not optimal crystal structures or composition ratios.

**Figure 3:**
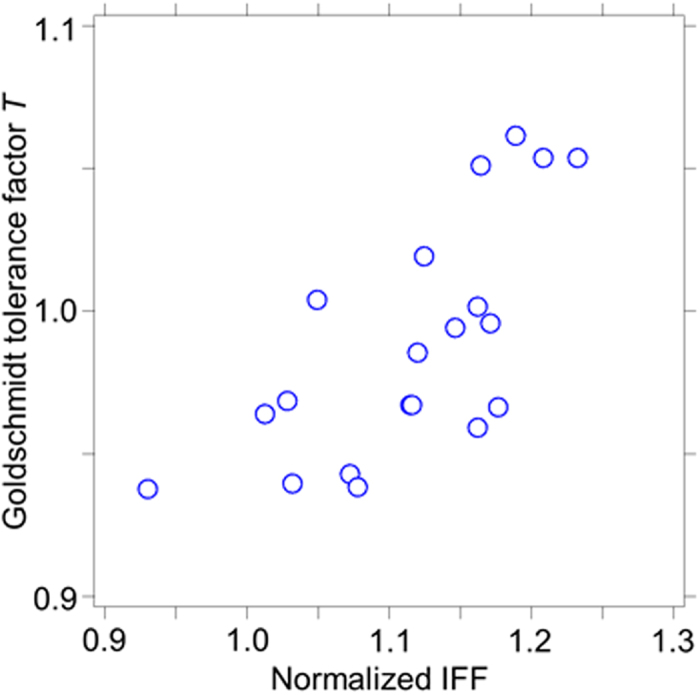
Relationship between the *T* and the IFF. The *T*, as a function of normalized IFF for the perovskite oxides (blue open circles (a total of 20 perovskite compounds)).

The *T* = 1 for the ideal perovskite oxides ABO_3_ is shown in a plot of *V*_unit_/*Z* vs. *V*_ion_ in Fig. 2. Here, the lattice constant *a* is set as the sum of the diameters of B ions and oxygen ions (radius: 1.40 Å), and the ratio between *R*_A_ and *R*_B_ is estimated from the *T*. It is worth noting that the data for the crystals with *T* = 1.0 (blue line) is distributed in the vicinity of the *V*_unit_/*Z* = 1/0.69 × *V*_ion_ line (black line). In addition, considered on the relationship between the *T* and the IFF on the perovskite oxides listed in the supplementary Table 2 in the Supplementary information, the *T* as a function of normalized IFF by the constant IFF for perovskite oxides shows in Fig. 3. The normalized IFF indicates a deviation from the constant IFF value. The values of the *T* show to increase with increasing of their IFF (tightly ion packed crystal structure) as described above on an ideal cubic perovskite compound (c.f. Fig. 1b). Therefore the IFF can be consistent with the *T*. This suggests that we expect to extend the applicability of the *T* concept for different ionic compounds using IFF. Thus, the IFF is available for evaluation of crystal structures with arbitrary ionic arrangements and compositions.

### Crystal structure analysis of novel hydride compounds using the IFF

**Figure 4:**
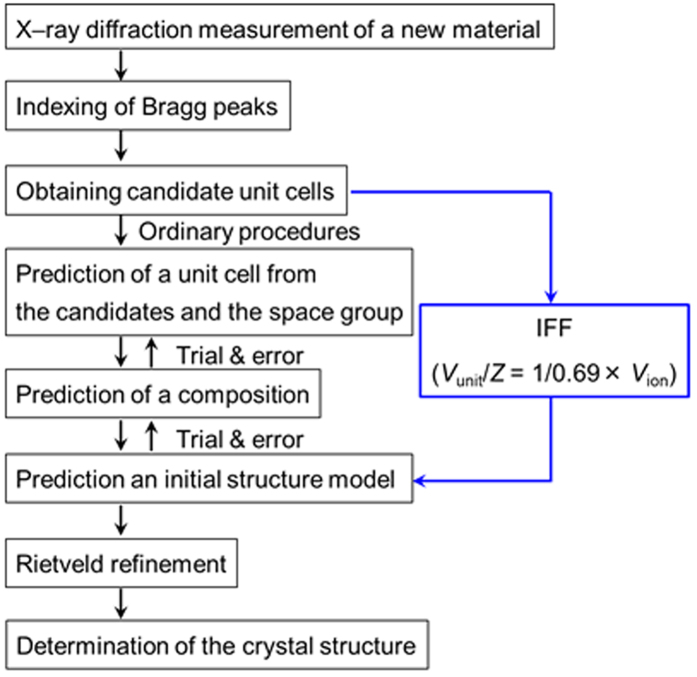
The flow chart for crystal structure determination. The general procedure follows by black arrows. Using the IFF, the selection of an optimal unit cell and composition ratio is simplified.

The selection of an optimal unit cell and composition ratio is one of the most difficult steps in the determination of the crystal structures. However, this can be considerably simplified by using the linearity obtained from the IFF and following the procedure summarized in the flow chart of Fig. 4. As an example, we demonstrate the use of this method by determining the crystal structure of three Al–based complex, LiCa(AlH_4_)_3_, LiCaAlH_6_ and Sr(AlH_4_)Cl, all showing new crystal structure and adopting complicated crystal structures where both mixed cations or anions coexist.

### Crystal structure analysis of LiCa(AlH_4_)_3_ containing different metallic cations

**Figure 5:**
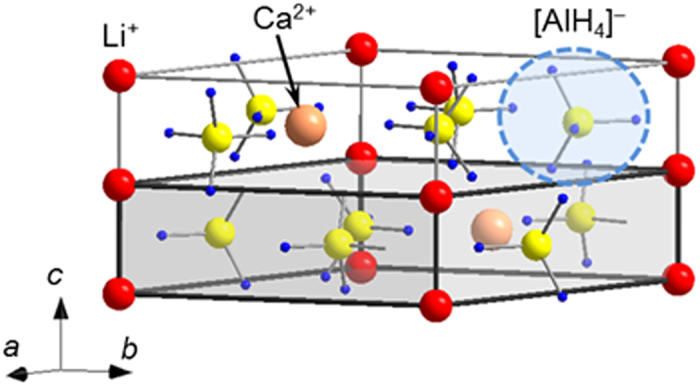
Crystal structure of LiCa(AlH_4_)_3_. The red, beige, yellow, blue and light-blue spheres, as well as the grey cuboid represent the Li^+^, Ca^2+^, Al, H, [AlH_4_]^−^ and the *V*_unit_/*Z* of LiCa(AlH_4_)_3_, respectively.

Considering a molar ratios of 1:1:3 for the starting materials LiH, CaH_2_ and AlH_3_, and evaluating the Raman and infrared spectra (Supplementary Fig. 1) and the powder X-ray diffraction (PXD) pattern (Supplementary Fig. 2), we assumed the formation of a compound with stoichiometry LiCa(AlH_4_)_3_. Indexing of the Bragg peaks derived from the diffraction patterns of LiCa(AlH_4_)_3_ shows that there are several possible candidates for the unit cell. This is likely due to poor crystallinity of the sample and the overlap of Bragg peaks from multiple phases or the same phase, and thus a limited number of Bragg peaks that can be used for indexing. Generally, an optimal unit cell is selected out of these candidates by means of trial and error and the space group, composition ratio and initial structure model of the lattice must be predicted. The use of the linearity obtained from the IFF minimizes this trial and error process (Fig. 4). Having already anticipated the composition of the new phase to be LiCa(AlH_4_)_3_, we can estimate the *V*_ion_ by using the ionic radii for Li^+^, Ca^2+^ and [AlH_4_]^−^ (Li^+^ = 0.59–0.92 Å; Ca^2+^ = 1.00–1.34 Å; [AlH_4_]^−^ = 2.26 Å). This results in a value between 150 and 158 Å^3^. Using the relationship *V*_unit_/*Z* = 1/0.69 × *V*_ion_, *V*_unit_/*Z* was estimated to be in the 217–230 Å^3^ range. Since *Z* is an integer, *V*_unit_ will roughly be an integer multiple of *V*_unit_/*Z*. By searching for unit cells with the appropriate indices and a *V*_unit_ value close to an integer multiple of this *V*_unit_/*Z*, the range of the possible unit cells is narrowed down and the selection of the candidate unit cell is simplified. In this work, the unit cell of LiCa(AlH_4_)_3_ was chosen to be a hexagonal crystal (*a* ≈ 8.92 Å, *c* ≈ 5.90 Å, *V*_unit_ ≈ 406 Å^3^) with *Z* = 2. Based on *hkl* extinction rules, we predicted the space group to be either *P*6_3_ (No. 173), *P*6_3_/*m* (No. 176) or *P*6_3_22 (No. 182), and using this information, we then identified the CdTh(MoO_4_)_3_ structure^34^ (space group *P*6_3_/*m* (No. 176), *a* = 9.80 Å, *c* = 6.35 Å) as the optimal initial crystal structure model. The complete crystal structure of LiCa(AlH_4_)_3_ was then determined by combining the Rietveld refinement fit (Supplementary Fig. 3) with first–principles calculations (Fig. 5). In the crystal structure determination, the hydrogen atomic positions are determined from first–principles calculations. Supplementary Table 15 and Supplementary Fig. 4 show the atomic positions and the total electronic density of states obtained from first–principles calculations, respectively.

In the LiCa(AlH_4_)_3_ crystal structure, Li^+^ is six–fold coordinated by [AlH_4_]^−^, Ca^2+^ is nine–fold coordinated by [AlH_4_]^−^ and [AlH_4_]^−^ is two–fold coordinated by Li^+^ and three–fold coordinated by Ca^2+^; thus, the total CN = 5 (detailed inter–atomic distances are shown in the Supplementary Table 16). The [AlH_4_]^−^ coordination number is in between the coordination of [AlH_4_]^−^ in the related compounds LiAlH_4_ (CN = 4)^9^ and Ca(AlH_4_)_2_ (CN = 6)^35^.

Recently, the crystal structure of LiCa(AlH_4_)_3_ was reported based on experiments^36^ and theoretical calculations^37^. While our crystal structure model has the same unit cell parameters and space group as those previously reported, our model has a different atomic arrangement. In the crystal structure proposed here, Li^+^ is located between Ca^2+^ and [AlH_4_]^−^, in contrast with the previously reported model, where all ions are positioned on the same (004) plane. From Rietveld refinements it is showed that the fit of the previously reported model is worse (*R*_*wp*_ = 0.046) than ours (*R*_*wp*_ = 0.040). First–principles calculations also showed that the previously reported model is less stable than our crystal structure model, the difference being 36 kJ/mol. Furthermore, and phonon calculations show the presence of imaginary frequencies. These results suggest the model we propose is a more accurate description of the crystal structure.

### Crystal structure analysis of the new hydride LiCaAlH_6_ containing different metallic cations

**Figure 6:**
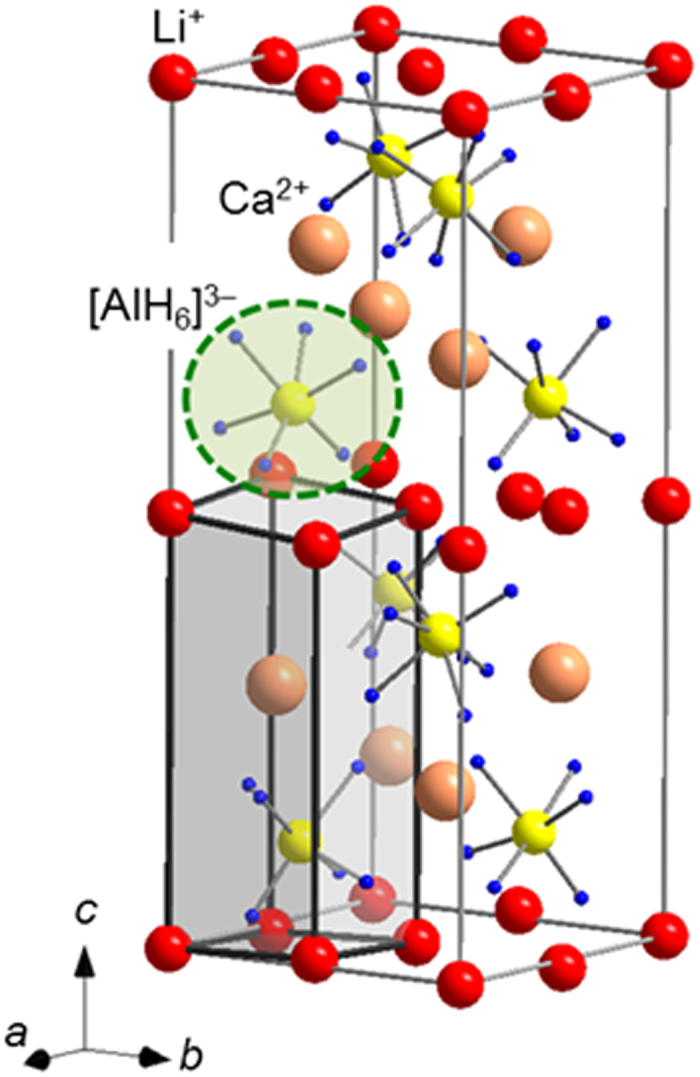
Crystal structure of LiCaAlH_6_. The red, beige, yellow, blue and light-green spheres, as well as the grey cuboid represent the Li^+^, Ca^2+^, Al, H, [AlH_6_]^3−^ and the *V*_unit_/*Z* of LiCaAlH_6_, respectively.

LiCa(AlH_4_)_3_ releases hydrogen at around 400 K (the thermogravimetric curve at 5 K/min in a He gas flow of 150 ml/min is shown in Supplementary Fig. 5). The PXD of a sample after hydrogen release is shown in Supplementary Fig. 2. Although it was difficult to observe the characteristic vibrational modes of [AlH_6_]^3−^ by Raman or infrared spectroscopies, we expected the formation of LiCaAlH_6_ after hydrogen release from LiCa(AlH_4_)_3_, in analogy with the related mixed cation alanate LiMg(AlH_4_)_3_^6^. Accordingly, we can estimate the *V*_ion_ by using the ionic radii for Li^+^, Ca^2+^ and [AlH_6_]^3−^ (Li^+^ = 0.59–0.92 Å; Ca^2+^ = 1.00–1.34 Å; [AlH_6_]^3−^ = 2.56 Å). This results in a value between 75 and 84 Å^3^. Using the relationship *V*_unit_/*Z* = 1/0.69 × *V*_ion_, the *V*_unit_/*Z* was estimated to be in the 109–121 Å^3^ range. The PXD pattern of LiCaAlH_6_ is indexed using a primitive tetragonal unit cell with *a* ≈ 6.59 Å, *c* ≈ 16.78 Å, and *V*_unit_ ≈ 729 Å^3^. *Z* is estimated to be 6 or 7 from the estimated *V*_ion_, but hydrides with [AlH_6_]^3−^ are plotted in the region of *V*_unit_/*Z *≦ 1/0.69 × *V*_ion_ in Fig. 2. This suggests that hydrides with [AlH_6_]^3−^ adopts tightly ionic packed crystal structures. Therefore, the range of *Z* is expanded to being in the range of 6–8. Since no prototype structure was found from the unit cell (tetragonal), the *Z* (=6–8) and the composition, the initial crystal structure model was predicted in combination with ab initio structural determination FOX^38^. Using Rietveld analysis and first-principles calculations, the crystal structure in the space group: *P*–4 (No. 81) was finally determined. The Rietveld fit is shown in Supplementary Fig. 6. This new crystal structure is shown in Fig. 6. In the crystal structure determination, the Li and H atomic positions are determined from first–principles calculations. Supplementary Table 17 and Supplementary Fig. 7 show the atomic positions and the total electronic density of states obtained from first–principles calculations, respectively. The value of *Z* and IFF are finally determined as 8 and 0.82 (*V*_ion_ = 75 Å^3^ and *V*_unit_/*Z* = 91 Å^3^), respectively. The IFF agrees reasonably with hydrides with [AlH_6_]^3−^ (See Fig. 2 and the Supplementary Table 14).

In the crystal structure, Li^+^ is four–fold coordinated by [AlH_6_]^3−^, Ca^2+^ is five–fold coordinated by [AlH_6_]^3−^ and [AlH_6_]^3−^ is four–fold coordinated by Li^+^ and five–fold coordinated by Ca^2+^ (the selected metal–hydrogen inter-atomic distances are listed in Supplementary Table 18). The CN around [AlH_6_]^3−^ (or the AlH6 unit) is 12 in the related Li_3_AlH_6_^9^ and 6 or 7 in CaAlH_5_^35^. The CN around [AlH_6_]^3−^ in LiCaAlH_6_ was found to be 9, which is the mean of the CNs in Li_3_AlH_6_ and CaAlH_5_. This is similar to LiCa(AlH_4_)_3_.

### Crystal structure analysis of the new hydride Sr(AlH_4_)Cl containing different anions

**Figure 7:**
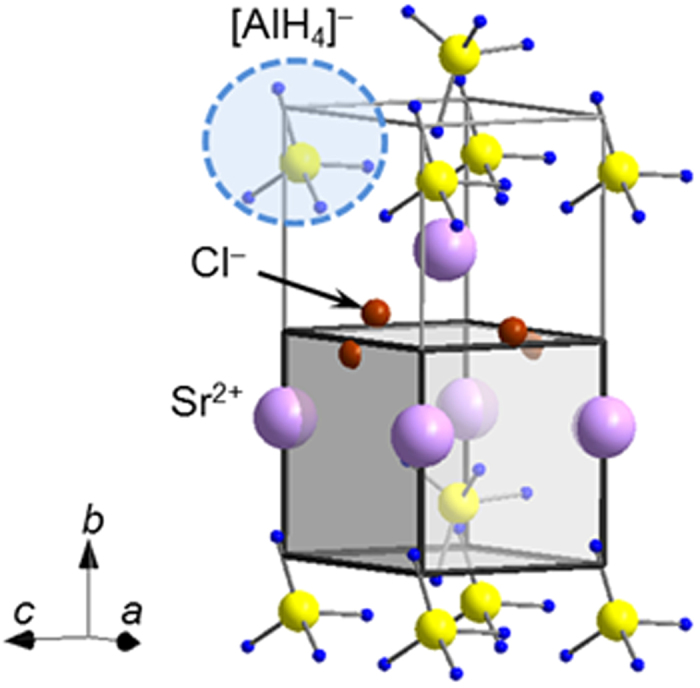
Crystal structure of Sr(AlH_4_)Cl. Purple, yellow, blue, light-blue and brown spheres and grey cuboids represent Sr^2+^, Al, H, [AlH_4_]^−^, Cl^−^ and *V*_unit_/*Z* of Sr(AlH_4_)Cl, respectively.

The sample was synthesized by the solid-state metathesis reaction of *x*LiAlH_4_ + SrCl_2_ (*x* = 1.0–3.0), with *x* = 1.3 providing the highest sample purity (Supplementary Fig. 8). The Bragg peaks observed in the PXD patterns of the as-prepared sample allowed indexing with an orthorhombic unit cell (*a* ≈ 5.23 Å, *b* ≈ 9.10 Å, *c* ≈ 4.32 Å, *V*_unit_ ≈ 206 Å^3^). The space groups was predicted to be either *Pmn*2_1_ (No. 31) or *Pmmn* (No. 59) based on the *hkl* extinction rules. Raman and Fourier transform infrared spectra (Supplementary Fig. 9) proved that the sample contained [AlH_4_]^−^ complex anions (tetra–alanates). Since the *V*_unit_/*Z* of all tetra–alanates is bigger than 69 Å^3^ (Supplementary Table 13)^9^, the *Z* value of the target sample is expected to be 1–3. When *Z* = 1 (*V*_unit_/*Z* ≈ 206 Å^3^), we expect 3 moles of [AlH_4_]^−^ anions per formula unit, which must be counter-balanced by three positive charges from the cation(s) (Supplementary Table 13). One possible candidate is LiSr(AlH_4_)_3_ with a hexagonal unit cell (*a* = 8.9269 Å and *c* = 5.8941 Å, *V*_unit_/*Z* ≈ 220 Å^3^), which is however identified in the PXD pattern of the starting mixture 3.0LiAlH_4_ + SrCl_2_ (Supplementary Fig. 8). In the PXD pattern of the starting mixture 3.0LiAlH_4_ + SrCl_2_, the new phase and LiSr(AlH_4_)_3 _can be clearly distinguished. Thus we have to exclude *Z* = 1. *Z* = 3 results in a too small *V*_unit_/*Z* ≈ 69 Å^3^, which is the same value observed for LiAlH_4_ (*V*_unit_/*Z* ≈ 69 Å^3^)^9^ and the smallest *V*_unit_/*Z* in the tetra–alanates. We must therefore also rule out *Z* = 3. This leaves *Z* = 2 as the only possible value, resulting in *V*_unit_/*Z* ≈ 103 Å^3^. The composition ratio that fulfils the conditions of IFF < 1.0 and electro–neutrality is predicted to be Sr(AlH_4_)Cl (80 Å^3^ < *V*_ion_ < 86 Å^3^, 0.78 < IFF < 0.84), with coexisting [AlH_4_]^−^ and Cl^−^ anions. Based on this information, the optimal initial crystal structure model was found isostructural with the PbCl(ReO_4_) structure (space group *Pmn*2_1_ (No. 31), *a* = 5.68 Å, *b* = 9.44 Å and *c* = 4.47 Å)^39^. Owing to the difficulties in distinguishing Al and Cl with close number of electrons and atomic positions of H by PXD, the crystal structure was eventually determined by combining synchrotron radiation powder X–ray (SR–PXD) and neutron diffraction (PND) diffraction of the deuteride analogue. The crystal structure of Sr(AlH_4_)Cl is shown in Fig. 7. The Rietveld refinement fit and detailed atomic positions are shown in Supplementary Figs 10 and 11 and Supplementary Table 19, respectively.

The results of crystal structure analysis revealed a novel Sr(AlH_4_)Cl compound with coexisting [AlH_4_]^−^ and Cl^−^ anions. In this structure, Sr^2+^ is coordinated by four Cl^−^ and five [AlH_4_]^−^ ions (detailed inter–atomic distances are shown in Supplementary Table 20). So far there have been no reports on tetra–alanates that contain different anions coexisting within the same structure, but borohydrides with coexisting [BH_4_]^−^ and Cl^−^ anions are reported^40^. Therefore, the results of this study suggest that the replacement of [AlH_4_]^−^ with halide ions is possible also in tetra–alanates, leading to novel tetra–alanates with potentially different material properties.

The possibility of using the IFF for determining the thermochemical radii or for assessing metal-hydrogen bonds, besides crystal structure analysis, is discussed in greater details in the Supplementary Information.

## Discussion

Ionic compounds with arbitrary ionic arrangements and compositional ratios were sorted using the unit cell volume per formula unit (*V*_unit_/*Z*) and the total volume of spherical ions (*V*_ion_) estimated through stoichiometry assuming spherical constituent ion in order to extend the applicability of Goldschmidt tolerance factor (*T*). The ratio between *V*_unit_/*Z* and *V*_ion_ defined as the ionic filling fraction (IFF) was used to extract ionic packing information from ionic compounds similar to using *T*. The IFF for most ionic compounds are interestingly distributed around a line given by *V*_unit_/*Z* = 1/0.69 × *V*_ion_. The linearity (the inverse of the slope) suggests that ionic compounds adopt a constant IFF value (0.69). The linearity was also consistent with the ideal cubic perovskite structure with the *T* = 1. Furthermore, the IFF showed the trend that the IFF increases with increasing *T*. Thus, the IFF allows uniformly classifying the data for a variety of ionic compounds and extending the applicability of the *T* concept for arbitrary ionic compounds.

The use of this linear relationship facilitates the selection of unit cell and compositional composition ratios for novel hydrides, which has often been a bottleneck for the determination of their crystal structures. We have been able to elucidate three new crystal structures, LiCa(AlH_4_)_3_, LiCaAlH_6_ and Sr(AlH_4_)Cl, respectively, with different cations and anions coexisting within the crystal structures. Therefore, the IFF could become a new indicator for crystal structure determination.

## Methods

### Synthesis

LiCa(AlH_4_)_3_ was directly synthesized by mechanochemical milling of LiH (Alfa Aesar, 99.4%), CaH_2_ (Sigma-Aldrich, 99.99%) and AlH_3_ in a molar ratios of 1:1:3. AlH_3_ as the starting material was synthesized in diethyl ether according to the chemical reaction of LiAlH_4_ and AlCl_3_^41^,^42^. The mixture was ball milled at 400 r.p.m. under a hydrogen gas pressure of 0.2 MPa using Fritsch P7. The effective milling time to obtain LiCa(AlH_4_)_3_ was 10 hours. Milling time of 15 min was alternated with pauses of 5 min duration.

LiCaAlH_6_ was yielded from LiCa(AlH_4_)_3_ which was kept at 400 K for 1 hour in 0.10 MPa of Ar gas pressure.

Sr(AlH_4_)Cl was synthesized by mechanochemical milling of LiAlH_4_ (Sigma–Aldrich, 95%) and SrCl_2_ (Sigma-Aldrich, 99.9%) in a molar ratios of *x*:1 (*x* = 1.0–3.0). The mixture was milled at 400 rpm under an Ar atmosphere of 0.10 MPa using Fritsch P7. The effective milling time was 5 h. Milling time of 15 min was alternated with pauses of 5 min duration.

Sr(AlD_4_)Cl for powder synchrotron radiation (SR-PXD) X-ray and neutron diffraction (PND) was synthesized by mechanochemical milling of LiAlD_4_ (ACROS Organic, 93%) and SrCl_2_ (Sigma-Aldrich, 99.9%) in a molar ratios of 1.3:1.

### X–ray diffraction (PXD)

All samples were initially measured by a conventional powder X–ray diffractometer (PANalytical X’PERT, with Cu Kα radiation (wavelength λ = 1.5406 Å for Kα1 and 1.5444 Å for Kα2)) at room temperature. The samples of LiCa(AlH_4_)_3_ and LiCaAlH_6_ for PXD and the Rietveld refinements were placed in Lindemann glass capillary (outside diameter = 0.50 mm, thickness = 0.01 mm) and sealed with paraffin liquid for the powder X-ray diffraction measurement with a transmission geometry at room temperature.

### High–resolution synchrotron radiation X–ray diffraction (SR–PXD)

The high-resolution SR–PXD data of Sr(AlD_4_)Cl were collected at room temperature at the Swiss-Norwegian beamlines (station BM01B) at the European Synchrotron Radiation Facility (ESRF) in Grenoble, France. The sample was placed in a rotating 0.5 mm boron-silica glass capillary. The wavelength 0.50513 Å was obtained from a channel-cut Si (111) monochromator. Data was collected up to 40° in step of 0.0065° in 2θ.

### Powder neutron diffraction (PND)

PND data of Sr(AlD_4_)Cl were collected at room temperature with the PUS instrument at the JEEP II reactor at Kjeller, Norway. The sample was placed in a cylindrical vanadium sample holder with a diameter of 6 mm. The wavelength 1.5539 Å was obtained from a Ge (511) focusing monochromator. Data was collected from 10° to 130° in step of 0.05° in 2*θ*.

### Raman and infrared spectroscopies

The Raman spectra of LiCa(AlH_4_)_3_ and Sr(AlH_4_)Cl were obtained by Nicolet Almega–HD with a Nd:YVO_4_ laser (532 nm). The sample was placed under Ar gas in a sample holder with a glass window, and spectra were measured using the backscattering configuration with a microscope Raman spectrometer. The FTIR spectra of LiCa(AlH_4_)_3_ and Sr(AlH_4_)Cl were obtained by Thermo Scientific Nicolet iZ10. A thin sample, approximately a few μm thick, was prepared in an Ar–gas–filled diamond anvil cell, and transmission spectra were measured with a microscope FTIR spectrometer.

### Thermal analysis

The hydrogen release reaction of LiCa(AlH_4_)_3_ was studied by thermogravimetry (TG, Rigaku TG-8210) with an Al sample holder heated up to 673 K at 5 K/min in a He gas flow of 150 ml/min.

### Crystal structural determination

The X-ray diffraction peaks of LiCa(AlH_4_)_3_, LiCaAlH_6_ and Sr(AlD_4_)Cl were indexed by TREOR97^43^ and PIRUM^44^. Structural determinations of LiCa(AlH_4_)_3_ and LiCaAlH_6_ based on PXD data and Sr(AlD_4_)Cl with SR-PXD and PND data were performed combined with the ab initio structural determination program FOX (version 1.9.0.2)^38^, the Rietveld program GSAS with the graphical interface EXPGUI (version 1.80)^45^ and first–principles calculations. In the Rietveled analysis, the Pseudo-Voigt peak shape function with the Finger–Cox–Jephcoat asymmetry correction^46^,^47^ was used. The background was modeled by Chebyschev polynomial function model in GSAS.

All samples were handled in Ar or He gas filled glove boxes with a dew point below 183 K and with less than 1 ppm of O_2_ to prevent (hydro–) oxidation.

### First–principles calculations

The crystal structures of LiCa(AlH_4_)_3_ and LiCaAlH_6_ were studied using first-principles calculations based on density-functional theory (DFT). We used a plane-wave basis and the projector augmented wave method^48^ within the generalized gradient approximation of Perdew and co-workers^49^, as implemented in vienna ab initio simulation package (VASP)^50^,^51^,^52^. To begin with, the experimentally determined structures of LiCa(AlH_4_)_3_ and LiCaAlH_6_ were fully relaxed with placing the H atoms to coordinate Al in the tetrahedral and octahedral fashions, respectively. After the relaxations, phonon calculations were performed to verify if the relaxed structures were at the true minimums. When imaginary phonon frequencies were observed, we slightly displaced the atoms along the directions of eigenvectors of the imaginary modes and further relaxed the structures to eliminate them. This procedure was carried out until the ground states were reached, in the same manner as in the prediction of crystal structures for several new complex transition-metal hydrides^13^. This was done using well-converged plane-wave basis sets with cut-off energies of 800 eV and 600 eV for LiCa(AlH_4_)_3_ and LiCaAlH_6_, respectively. The Brillouin-zone samplings were performed using the special k-point method^53^ with 4 × 4 × 8 and 8 × 8 × 4 meshes for LiCa(AlH_4_)_3_ and LiCaAlH_6_, respectively.

## Additional Information

**How to cite this article**: Sato, T. *et al*. Extending the applicability of the Goldschmidt tolerance factor to arbitrary ionic compounds. *Sci. Rep.* **6**, 23592; doi: 10.1038/srep23592 (2016).

## Supplementary Material

Supplementary Information
