# Supplementary material for: Extending the applicability of the Goldschmidt tolerance factor to arbitrary ionic compounds
Source: Sci Rep. 2016 Apr 1;6:23592. doi: 10.1038/srep23592 (PMC4817026; doi:10.1038/srep23592)
Supplement: Supplementary Information [file srep23592-s1.doc]

Supplementary Information

Extending the applicability of the Goldschmidt tolerance factor to arbitrary ionic compounds

Toyoto Sato1, Shigeyuki Takagi1, Stefano Deledda2, Bjørn C. Hauback2, and Shin-ichi Orimo1,3,*

1Institute for Materials Research, Tohoku University, Aoba-ku, Sendai 980-8577, Japan

2Institute for Energy Technology, Physics Department, Kjeller, NO-2027, Norway

3WPI-Advanced Institute for Materials Research, Tohoku University, Sendai 980-8577, Japan

Corresponding. [orimo@imr.tohoku.ac.jp](mailto:orimo@imr.tohoku.ac.jp)

**Table of Contents**

**1. Definition of thermochemical radius and coordination numbers (CNs)**

**2. Supplementary Tables**

- List of *V*unit/*Z* and *V*ion of oxides
- List of *V*unit/*Z* and *V*ion of perovskite–type oxides
- List of *V*unit/*Z* and *V*ion of fluorides
- List of *V*unit/*Z* and *V*ion of chlorides
- List of *V*unit/*Z* and *V*ion of bromides
- List of *V*unit/*Z* and *V*ion of iodides
- List of *V*unit/*Z* and *V*ion of binary hydrides
- List of *V*unit/*Z* and *V*ion of perovskite–type oxides
- List of *V*unit/*Z* and *V*ion of borohydrides
- List of *V*unit/*Z* and *V*ion of imides
- List of *V*unit/*Z* and *V*ion of amides
- List of *V*unit/*Z* and *V*ion of mixed [BH4]– and [NH2]– complex hydrides
- List of *V*unit/*Z* and *V*ion of tetra–alanates
- List of *V*unit/*Z* and *V*ion of hexa–alanates
- Crystallographic parameters of LiCa(AlH4)3
- Selected metal-hydrogen inter-atomic distances in LiCa(AlH4)3
- Crystallographic parameters of LiCaAlH6
- Selected metal-hydrogen inter-atomic distances in LiCaAlH6
- Crystallographic parameters of Sr(AlD4)Cl
- Selected metal-hydrogen inter-atomic distances in Sr(AlD4)Cl

**3. Supplementary Figures**

- Raman and FTIR spectra of LiCa(AlH4)3
- X-ray diffraction pattern of LiCa(AlH4)3 and LiCaAlH6
- The Rietveld refinement fit of PXD for LiCa(AlH4)3
- Density of state for LiCa(AlH4)3
- The Rietveld refinement fit of PXD for LiCaAlH6
- Density of state for LiCaAlH6
- X-ray diffraction pattern of *x*LiAlH4 + SrCl2 (*x* = 1.0 – 3.0)
- Raman and FTIR spectra of Sr(AlH4)Cl and Sr(AlD4)Cl
- The Rietveld refinement fit of SR–PXD for Sr(AlD­4)Cl
- The Rietveld refinement fit of PND for Sr(AlD­4)Cl

**4. Estimation of thermodynamical radii of complex anions**

**5. Bonding nature of Al – H in AlH3**

**6. Supplementary References**

**1. Definition of thermochemical radius1–4 and coordination numbers (CNs)**

The thermochemical radius was defined by equation 1.

(Equation 1)

*rc*: Thermo-chemical radius of cation (in nm)

*ra*: Thermo-chemical radius of anion (in nm)

*ν*: Number of element (e.g. 2 for NaCl)

*Za*: modulus of the anion charge

*Zc*: modulus of the cation charge

*UPOT*: Lattice energy of a crystal (in kJ/mol; e.g. LiBH4: 778 kJ/mol4)

If a Shannon radius for a particular coordination number (CN) does not exist, the target Shannon radius is estimated using linear interpolation of the relationship between the CN and the Shannon radii. In this work, To estimate the CN of cations in complex hydrides, we considered the whole complex anion as its ligand. For example, in LiBH4 the ionic radius of Li+ is 0.59 Å (CN = 4), whereas the radius of [BH4]− , estimated from equation (1), is 2.05 Å. This results in an ionic volume *V*ion for LiBH4 of 4/3 × π × {(0.59)3 + (2.05)3} = 36.95 Å3.

**2. Supplementary Tables**

**Supplementary Table 1** List of *V*unit/*Z* and *V*ion of oxides5 – 12

| Compounds | *V*unit/*Z* (Å3) | *V*ion (Å3) | *V*ion/*V*unit/*Z* |
| --- | --- | --- | --- |
| Li2O | 24.70 | 13.71 | 0.56 |
| MgO | 18.69 | 14.95 | 0.80 |
| Al2O3 | 42.57 | 34.31 | 0.81 |
| TiO2 | 31.21 | 22.00 | 0.70 |
| MgAl2O4 | 65.94 | 46.09 | 0.70 |
| Y3Fe5O12 | 236.95 | 149.12 | 0.63 |
| MoO2 | 30.02 | 12.35 | 0.41 |
| Mg3Al2(SiO4)3 | 188.04 | 86.26 | 0.46 |
| Ag2Cu2O3 | 92.59 | 38.55 | 0.42 |

**Supplementary Table 2** List of *V*unit/*Z*, *V*ion and Goldschmidt tolerance factor *T* of perovskite oxides13 – 31

| Compounds | *V*unit/*Z* (Å3) | *V*ion (Å3) | *V*ion/*V*unit/*Z* | *T* |
| --- | --- | --- | --- | --- |
| NaNbO3 | 60.76 | 46.83 | 0.77 | 0.97 |
| NaTaO3 | 60.70 | 46.83 | 0.77 | 0.97 |
| KNbO3 | 64.71 | 54.06 | 0.84 | 1.05 |
| KTaO3 | 63.44 | 54.96 | 0.85 | 1.05 |
| CaTiO3 | 55.92 | 45.49 | 0.81 | 0.97 |
| CaRuO3 | 56.70 | 45.56 | 0.80 | 0.96 |
| SrTiO3 | 59.64 | 47.92 | 0.80 | 1.00 |
| SrRuO3 | 60.55 | 47.99 | 0.79 | 0.99 |
| BaTiO3 | 64.35 | 52.89 | 0.82 | 1.06 |
| BaZrO3 | 73.79 | 53.53 | 0.73 | 1.00 |
| BaIrO3 | 65.83 | 52.99 | 0.80 | 1.05 |
| BaCeO3 | 85.11 | 54.72 | 0.64 | 0.94 |
| PbTiO3 | 63.37 | 49.27 | 0.78 | 1.02 |
| PbZrO3 | 71.29 | 49.90 | 0.70 | 0.96 |
| PbHfO3 | 70.11 | 49.84 | 0.71 | 0.97 |
| BiScO3 | 68.66 | 48.98 | 0.71 | 0.94 |
| BiFeO3 | 62.48 | 48.38 | 0.77 | 0.99 |
| LaTiO3 | 62.43 | 46.28 | 0.74 | 0.94 |
| LaNiO3 | 56.51 | 45.75 | 0.81 | 1.00 |
| LaRuO3 | 62.19 | 46.34 | 0.75 | 0.94 |

**Supplementary Table 3** List of *V*unit/*Z* and *V*ion of fluorides32–36

| Compounds | *V*unit/*Z* (Å3) | *V*ion (Å3) | *V*ion/*V*unit/*Z* |
| --- | --- | --- | --- |
| LiF | 16.33 | 11.69 | 0.72 |
| NaF | 24.88 | 14.30 | 0.57 |
| KF | 38.22 | 20.86 | 0.55 |
| RbF | 45.13 | 24.56 | 0.54 |
| CsF | 54.38 | 29.36 | 0.54 |
| MgF2 | 32.61 | 19.97 | 0.61 |
| CaF2 | 40.78 | 24.72 | 0.61 |
| SrF2 | 48.78 | 27.21 | 0.56 |
| BaF2 | 59.48 | 30.83 | 0.52 |

**Supplementary Table 4** List of *V*unit/*Z* and *V*ion of chlorides32, 37–40

| Compounds | *V*unit/*Z* (Å3) | *V*ion (Å3) | *V*ion/*V*unit/*Z* |
| --- | --- | --- | --- |
| LiCl | 33.94 | 26.68 | 0.79 |
| NaCl | 44.86 | 29.28 | 0.65 |
| KCl | 62.31 | 35.85 | 0.58 |
| RbCl | 71.26 | 39.55 | 0.56 |
| CsCl | 70.09 | 46.91 | 0.67 |
| MgCl2 | 67.43 | 51.24 | 0.76 |
| CaCl2 | 84.26 | 53.87 | 0.64 |
| SrCl2 | 84.98 | 58.06 | 0.68 |
| BaCl2 | 87.64 | 62.98 | 0.72 |

**Supplementary Table 5** List of *V*unit/*Z* and *V*ion of bromides32, 40–43

| Compounds | *V*unit/*Z* (Å3) | *V*ion (Å3) | *V*ion/*V*unit/*Z* |
| --- | --- | --- | --- |
| LiBr | 41.62 | 33.38 | 0.80 |
| NaBr | 53.39 | 35.98 | 0.67 |
| KBr | 71.87 | 42.55 | 0.59 |
| RbBr | 81.74 | 46.25 | 0.57 |
| CsBr | 79.25 | 53.61 | 0.68 |
| MgBr2 | 79.35 | 64.64 | 0.81 |
| CaBr2 | 98.21 | 67.27 | 0.68 |
| SrBr2 | 96.83 | 71.46 | 0.74 |
| BaBr2 | 101.71 | 76.39 | 0.75 |

**Supplementary Table 6** List of *V*unit/*Z* and *V*ion of iodides32, 40, 44, 45

| Compounds | *V*unit/*Z* (Å3) | *V*ion (Å3) | *V*ion/*V*unit/*Z* |
| --- | --- | --- | --- |
| LiI | 54.62 | 46.44 | 0.85 |
| NaI | 67.80 | 49.05 | 0.72 |
| KI | 88.18 | 55.61 | 0.63 |
| RbI | 98.94 | 59.31 | 0.60 |
| CsI | 95.31 | 66.67 | 0.70 |
| MgI2 | 102.84 | 90.77 | 0.88 |
| CaI2 | 121.78 | 93.39 | 0.77 |
| SrI2 | 123.54 | 96.63 | 0.78 |
| BaI2 | 126.53 | 102.51 | 0.81 |

**Supplementary Table 7** List of *V*unit/*Z* and *V*ion of binary hydrides46–50

| Compounds | *V*unit/*Z* (Å3) | *V*ion (Å3) | *V*ion/*V*unit/*Z* |
| --- | --- | --- | --- |
| LiH | 16.75 | 13.33 | 0.80 |
| NaH | 29.23 | 15.94 | 0.55 |
| MgH2 | 30.63 | 24.55 | 0.80 |
| CaH2 | 36.34 | 29.87 | 0.82 |
| SrH2 | 45.03 | 32.40 | 0.72 |

**Supplementary Table 8** List of *V*unit/*Z* and *V*ion of perovskite-type hydrides51

| Compounds | *V*unit/*Z* (Å3) | *V*ion (Å3) | *V*ion/*V*unit/*Z* |
| --- | --- | --- | --- |
| LiSrH3 | 56.31 | 48.83 | 0.87 |
| LiBaH3 | 64.63 | 53.80 | 0.83 |
| LiEuH3 | 54.33 | 48.83 | 0.90 |
| NaMgH3 | 56.89 | 46.58 | 0.82 |
| RbMgH3 | 72.00 | 57.36 | 0.80 |
| RbCaH3 | 93.10 | 59.99 | 0.64 |
| CsMgH3 | 83.41 | 63.88 | 0.77 |
| CsCaH3 | 97.93 | 66.50 | 0.68 |

**Supplementary Table 9** List of *V*unit/*Z* and *V*ion of borohydrides52–75

| Compounds | *V*unit/*Z* (Å3) | *V*ion (Å3) | *V*ion/*V*unit/*Z* |
| --- | --- | --- | --- |
| LiBH4 | 54.62 | 36.95 | 0.68 |
| NaBH4 | 58.10 | 40.53 | 0.70 |
| KBH4 | 75.44 | 47.10 | 0.62 |
| RbBH4 | 86.33 | 50.80 | 0.59 |
| CsBH4 | 101.56 | 55.60 | 0.55 |
| α-Mg(BH4)2 | 113.71 | 72.95 | 0.64 |
| β-Mg(BH4)2 | 117.87 | 72.95 | 0.62 |
| γ-Mg(BH4)2 | 163.02 | 72.95 | 0.45 |
| α-Ca(BH4)2 | 106.88 | 76.36 | 0.71 |
| β-Ca(BH4)2 | 105.35 | 76.36 | 0.72 |
| γ-Ca(BH4)2 | 102.84 | 76.36 | 0.74 |
| Sr(BH4)2 | 110.73 | 79.06 | 0.71 |
| Mn(BH4)2 | 113.53 | 73.38 | 0.65 |
| LiK(BH4)2 | 122.99 | 86.07 | 0.70 |
| NaK(BH4)2 | 137.66 | 87.63 | 0.64 |
| Y(BH4)3 | 156.20 | 111.31 | 0.71 |
| Al(BH4)3 | 165.76 | 108.51 | 0.65 |
| NaZn(BH4)3 | 171.98 | 113.33 | 0.66 |
| KZn(BH4)3 | 184.43 | 119.89 | 0.65 |
| KCd(BH4)3 | 180.29 | 124.22 | 0.69 |
| Zr(BH4)4 | 201.23 | 145.21 | 0.72 |
| Hf(BH4)4 | 197.85 | 145.17 | 0.73 |
| LiSc(BH4)4 | 222.13 | 146.41 | 0.66 |
| NaSc(BH4)4 | 218.73 | 150.53 | 0.69 |
| KSc(BH4)4 | 234.09 | 164.56 | 0.70 |
| KY(BH4)4 | 256.41 | 158.41 | 0.62 |
| K2Mg(BH4)4 | 253.16 | 172.58 | 0.68 |
| K2Mn(BH4)4 | 250.90 | 174.40 | 0.70 |
| K2Cd(BH4)4 | 256.95 | 174.97 | 0.68 |
| LiZn2(BH4)5 | 295.65 | 182.54 | 0.62 |
| K3Mg(BH4)5 | 320.79 | 226.89 | 0.71 |
| NaZn2(BH4)5 | 328.01 | 185.75 | 0.57 |

**Supplementary Table 10** List of *V*unit/*Z* and *V*ion of imides76, 77

| Compounds | *V*unit/*Z* (Å3) | *V*ion (Å3) | *V*ion/*V*unit/*Z* |
| --- | --- | --- | --- |
| Li2NH | 32.45 | 10.77 | 0.33 |
| MgNH | 35.62 | 9.56 | 0.27 |

**Supplementary Table 11** List of *V*unit/*Z* and *V*ion of amides78

| Compounds | *V*unit/*Z* (Å3) | *V*ion (Å3) | *V*ion/*V*unit/*Z* |
| --- | --- | --- | --- |
| LiNH2 | 32.46 | 20.72 | 0.64 |
| NaNH2 | 47.14 | 23.93 | 0.51 |
| Mg(NH2)2 | 67.49 | 40.50 | 0.60 |

**Supplementary Table 12** List of *V*unit/*Z* and *V*ion of mixed [BH4]– and [NH2]– complex hydrides79

| Compounds | *V*unit/*Z* (Å3) | *V*ion (Å3) | *V*ion/*V*unit/*Z* |
| --- | --- | --- | --- |
| Li2(NH2)(BH4) | 90.24 | 57.67 | 0.64 |
| Li4(NH2)3(BH4) | 151.61 | 99.11 | 0.65 |

**Supplementary Table 13** List of *V*unit/*Z* and *V*ion of tetra-alanates80–82

| Compounds | *V*unit/*Z* (Å3) | *V*ion (Å3) | *V*ion/*V*unit/*Z* |
| --- | --- | --- | --- |
| LiAlH4 | 68.80 | 50.19 | 0.73 |
| NaAlH4 | 71.06 | 55.23 | 0.78 |
| KAlH4 | 94.47 | 61.39 | 0.65 |
| Mg(AlH4)2 | 136.81 | 98.27 | 0.72 |
| Ca(AlH4)2 | 144.57 | 102.59 | 0.71 |
| Sr(AlH4)2 | 103.07 | 106.12 | 1.03 |
| Eu(AlH4)2 | 100.91 | 105.91 | 1.05 |
| LiMg(AlH4)3 | 214.70 | 148.46 | 0.69 |
| LiCa(AlH4)3 | 203.48 | 153.78 | 0.76 |

**Supplementary Table 14** List of *V*unit/*Z* and *V*ion of hexa-alanates80, 83

| Compounds | *V*unit/*Z* (Å3) | *V*ion (Å3) | *V*ion/*V*unit/*Z* |
| --- | --- | --- | --- |
| Li3AlH6 | 89.45 | 72.86 | 0.81 |
| Na3AlH6 | 114.80 | 82.85 | 0.72 |
| Na2LiAlH6 | 100.69 | 80.24 | 0.80 |
| K2LiAlH6 | 124.94 | 92.92 | 0.74 |
| K2NaAlH6 | 133.75 | 96.26 | 0.72 |
| LiMgAlH6 | 80.61 | 72.34 | 0.90 |
| LiCaAlH6 | 91.16 | 74.73 | 0.82 |
| LaAlH6 | 77.74 | 76.81 | 0.99 |
| CeAlH6 | 75.51 | 76.53 | 1.01 |
| PrAlH6 | 73.69 | 76.26 | 1.03 |
| NdAlH6 | 72.65 | 75.99 | 1.05 |

**Supplementary Table 15** Crystallographic parameters for LiCa(AlH4)3 with *a* = 8.9269(4) Ǻ and *c* = 5.8941(3) Ǻ (*a* = 8.9278 Ǻ and *c* = 5.8947 Ǻ (first-principles calculations)) in the space group *P*63/*m* (No. 176) and *Z* = 2. The atomic positions obtained from first-principles calculations are given in parentheses. *Uiso* of all atoms are not refined.

| Atom | Wyckoff position | *x* | *y* | *z* | 100×*Uiso* | Occupancy |
| --- | --- | --- | --- | --- | --- | --- |
| Li | 2*b* | 0  (0) | 0  (0) | 0  (0) | 1.0 | 1.0  (1.0) |
| Ca | 2*c* | 1/3  (1/3) | 2/3  (2/3) | 1/4  (1/4) | 1.0 | 1.0  (1.0) |
| Al | 6*h* | 0.3819(3)  (0.3836) | 0.2827(3)  (0.2841) | 1/4  (1/4) | 1.0 | 1.0  (1.0) |
| H1 | 6*h* | (0.4434) | (0.4871) | (1/4) |  | (1.0) |
| H2 | 6*h* | (0.1710) | (0.1528) | (1/4) |  | (1.0) |
| H3 | 12*i* | (0.4548) | (0.2354) | (0.0242) |  | (1.0) |

**Supplementary Table 16** Selected metal-hydrogen inter-atomic distances in the crystal structure of LiCa(AlH4)3

|  | Distances (Ǻ) |
| --- | --- |
| Li–H2 (×6) | 2.0689 |
| Ca–H1 (×3)  Ca–H3 (×6) | 2.2607  2.3024 |
| Al–H1 (×1)  Al–H2 (×1)  Al–H3 (×2) | 1.6132  1.6588  1.6252 |

**Supplementary Table 17** Crystallographic parameters for LiCaAlH6 with *a* = 6.5940(1) Ǻ and *c* = 16.7965(5) Ǻ (*a* = 6.6652 Ǻ and *c* = 16.5607 Ǻ (first-principles calculations)) in the space group *P*–4 (No. 81) and *Z* = 8. The atomic positions obtained from first-principles calculations are given in parentheses. *Uiso* of all atoms are not refined.

| Atom | Wyckoff position | *x* | *y* | *z* | 100×*Uiso* | Occupancy |
| --- | --- | --- | --- | --- | --- | --- |
| Li1 | 1*a* | 0  (0) | 0  (0) | 0  (0) | 1.0 | 1.0  (1.0) |
| Li2 | 1*b* | 0  (0) | 0  (0) | 1/2  (1/2) | 1.0 | 1.0  (1.0) |
| Li3 | 1*c* | 1/2  (1/2) | 1/2  (1/2) | 0  (0) | 1.0 | 1.0  (1.0) |
| Li4 | 1*d* | 1/2  (1/2) | 1/2  (1/2) | 1/2  (1/2) | 1.0 | 1.0  (1.0) |
| Li5 | 2*g* | 0  (0) | 1/2  (1/2) | 0.5125(55)  (0.4843) | 1.0 | 1.0  (1.0) |
| Li6 | 2*g* | 0  (0) | 1/2  (1/2) | 0.0000(97)  0.0085 | 1.0 | 1.0  (1.0) |
| Ca1 | 4*h* | 0.2841(15)  (0.3119) | 0.2898(17)  (0.2730) | 0.1923(9)  (0.1937) | 1.0 | 1.0  (1.0) |
| Ca2 | 4*h* | 0.2208(14)  (0.2380) | 0.2276(18)  (0.1803) | 0.6912(8)  (0.6978) | 1.0 | 1.0  (1.0) |
| Al1 | 4*h* | 0.2267(24)  (0.2812) | 0.2816(20)  (0.2452) | 0.3744(8)  (0.3777) | 1.0 | 1.0  (1.0) |
| Al2 | 4*h* | 0.2597(23)  (0.2812) | 0.2506(29)  (0.2398) | 0.8795(7)  (0.8264) | 1.0 | 1.0  (1.0) |
| H1 | 4*h* | (0.4729) | (0.2445) | (0.3245) |  | (1.0) |
| H2 | 4*h* | (0.2947) | (0.0386) | (0.4346) |  | (1.0) |
| H3 | 4*h* | (0.1523) | (0.0958) | (0.2964) |  | (1.0) |
| H4 | 4*h* | (0.1623) | (0.4222) | (0.3053) |  | (1.0) |
| H5 | 4*h* | (0.2861) | (0.4340) | (0.4450) |  | (1.0) |
| H6 | 4*h* | (0.2449) | (0.0032) | (0.5840) |  | (1.0) |
| H7 | 4*h* | (0.0609) | (0.3014) | (0.8263) |  | (1.0) |
| H8 | 4*h* | (0.2532) | (0.4729) | (0.9283) |  | (1.0) |
| H9 | 4*h* | (0.3881) | (0.3663) | (0.7959) |  | (1.0) |
| H10 | 4*h* | (0.2891) | (0.0307) | (0.8161) |  | (1.0) |
| H11 | 4*h* | (0.2130) | (0.1039) | (0.9599) |  | (1.0) |
| H12 | 4*h* | (0.2158) | (0.4740) | (0.0859) |  | (1.0) |

**Supplementary Table 18** Selected metal-hydrogen inter-atomic distances in the crystal structure of LiCaAlH6

|  | Distances (Ǻ) |
| --- | --- |
| Li1-H11 (×4) | 1.7136 |
| Li2-H5 (×4) | 1.7485 |
| Li3-H2 (×4)  Li3-H6 (×4) | 2.2579  2.1444 |
| Li4-H8 (×4)  Li4-H12 (×4) | 2.0369  2.3751 |
| Li5-H2 (×2)  Li5-H5 (×2)  Li5-H6 (×2) | 1.9350  2.0624  2.0423 |
| Li6-H8 (×2)  Li6-H11 (×2)  Li6-H12 (×2) | 2.1553  2.1010  1.9338 |
| Ca1-H1 (×1)  Ca1-H3 (×1)  Ca1-H4 (×1)  Ca1-H7 (×1)  Ca1-H7 (×1)  Ca1-H8 (×1)  Ca1-H9 (×1)  Ca1-H9 (×1)  Ca1-H10 (×1)  Ca1-H12 (×1) | 2.4255  2.3288  2.3245  2.2514  2.9583  2.4806  2.2847  2.2945  2.2916  2.3219 |
| Ca2-H1 (×1)  Ca2-H2 (×1)  Ca2-H3 (×1)  Ca2-H3 (×1)  Ca2-H4 (×1)  Ca2-H4 (×1)  Ca2-H6 (×1)  Ca2-H7 (×1)  Ca2-H9 (×1)  Ca2-H10 (×1) | 2.3411  2.9641  2.2352  2.4128  2.2681  2.5935  2.2248  2.5645  2.2745  2.2247 |
| Al1-H1 (×1)  Al1-H2 (×1)  Al1-H3 (×1)  Al1-H4 (×1)  Al1-H5 (×1)  Al1-H6 (×1) | 1.7521  1.6995  1.7865  1.7714  1.7018  1.7761 |
| Al2-H7 (×1)  Al2-H8 (×1)  Al2-H9 (×1)  Al2-H10 (×1)  Al2-H11 (×1)  Al2-H12 (×1) | 1.7591  1.7623  1.7692  1.7436  1.6747  1.7380 |

**Supplementary Table 19** Crystallographic parameters for Sr(AlD4)Cl with *a* = 5.2241(4) Ǻ, *b* = 9.0851(7) Ǻ and *c* = 4.3229(3) Ǻ in the space group *Pmn*21 (No. 31) and *Z* = 2. Al–D distances in Sr(AlD4)Cl were soft constrained to 1.62 Å. The constraints were weighted so that they imposed only a minor deterioration of the fit to the experimental data.

| Atom | Wyckoff position | *x* | *y* | *z* | 100×*Uiso* | Occupancy |
| --- | --- | --- | --- | --- | --- | --- |
| Sr | 2*a* | 0 | 0.3076(3) | 0.9963(18) | 1.9(1) | 1.0 |
| Cl | 2*a* | 0.5 | 0.4701(3) | 0.0004(2) | 0.8(1) | 1.0 |
| Al | 2*a* | 0.5 | 0.1255(5) | 0.4195(7) | 0.6(2) | 1.0 |
| D1 | 2*a* | 0.5 | 0.1412(4) | 0.0462(7) | 2.8(1) | 1.0 |
| D2 | 2*a* | 0 | 0.0431(4) | 0.0416(18) | 2.8(1) | 1.0 |
| D3 | 4*b* | 0.2387(5) | 0.2126(4) | 0.5046(2) | 2.8(1) | 1.0 |

**Supplementary Table 20** Selected inter-atomic distances in the crystal structure of LiCa(AlH4)3

|  | Distances (Ǻ) |
| --- | --- |
| Sr–D1 (×2)  Sr–D2 (×1)  Sr–D3 (×2)  Sr–D3 (×2) | 3.026(3)  2.411(5)  2.611(9)  2.670(10) |
| Sr–Cl (×1)  Sr–Cl (×1)  Sr–Cl (×2) | 2.945(10)  2.971(10)  3.0003(20) |
| Al–D1 (×1)  Al–D2 (×1)  Al–D3 (×2) | 1.62003(24)  1.61999(23)  1.62003(17) |

**3. Supplementary Figures**


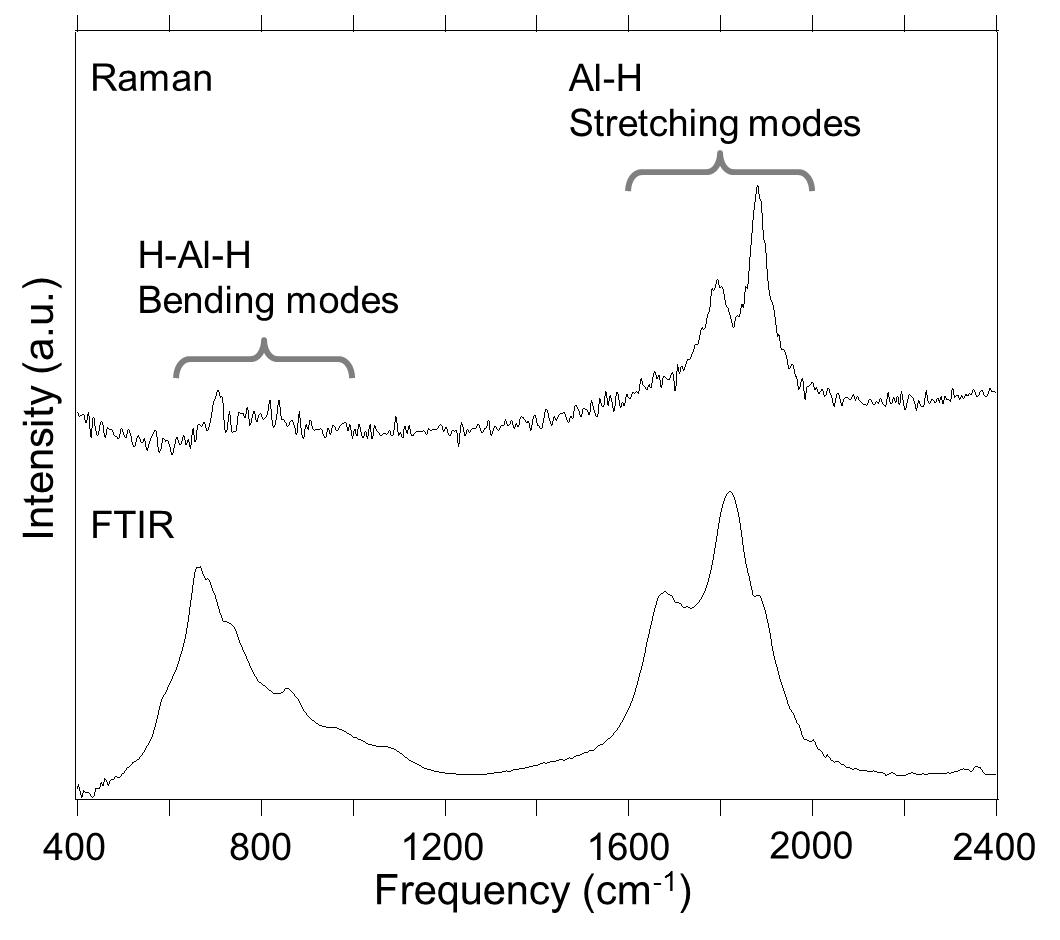


**Supplementary Figure 1** (top) Raman and (bottom) FTIR spectra of LiCa(AlH4)3. Compared with related tetra-alanate, NaAlH4,84 bending and stretching modes of [AlH4]– are assigned at around 700-900 and 1700-2000 cm–1, respectively.


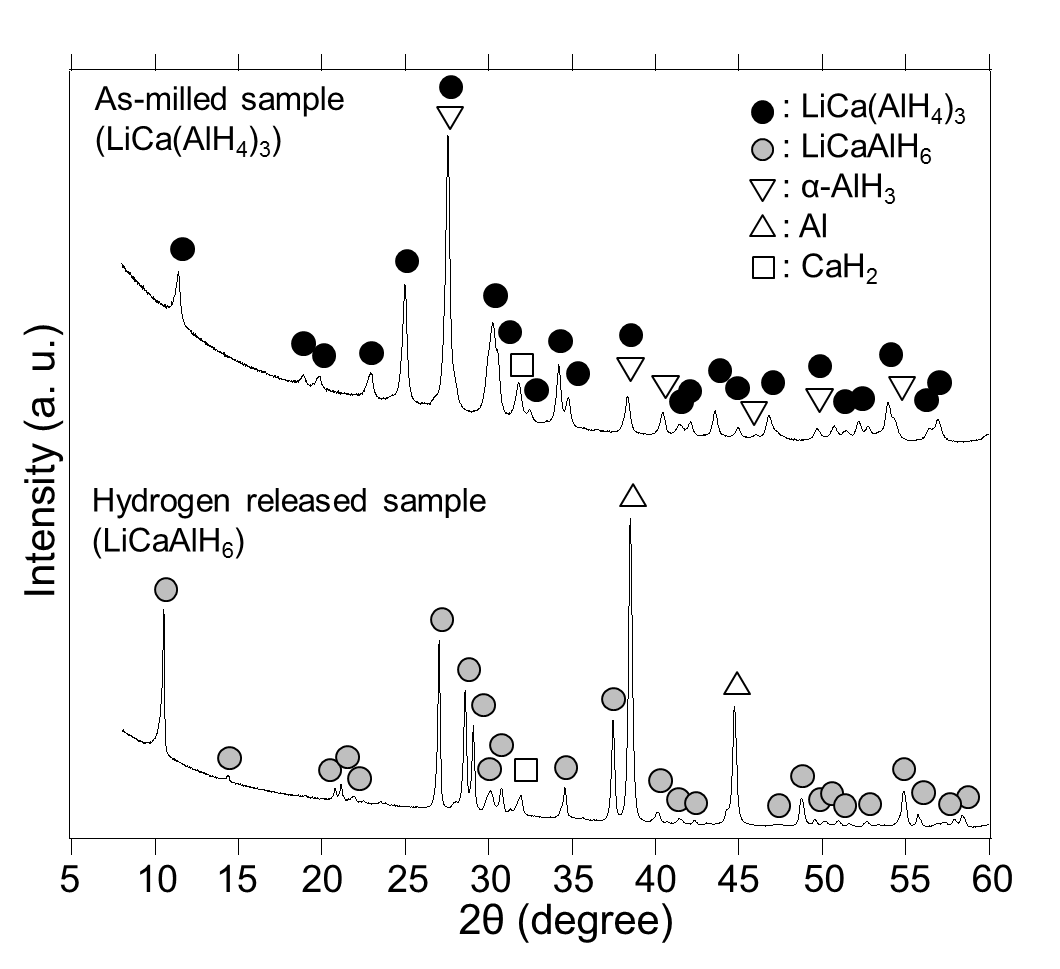


**Supplementary Figure 2** X-ray diffraction patterns of (top) LiCa(AlH4)3 and (bottom) LiCaAlH6


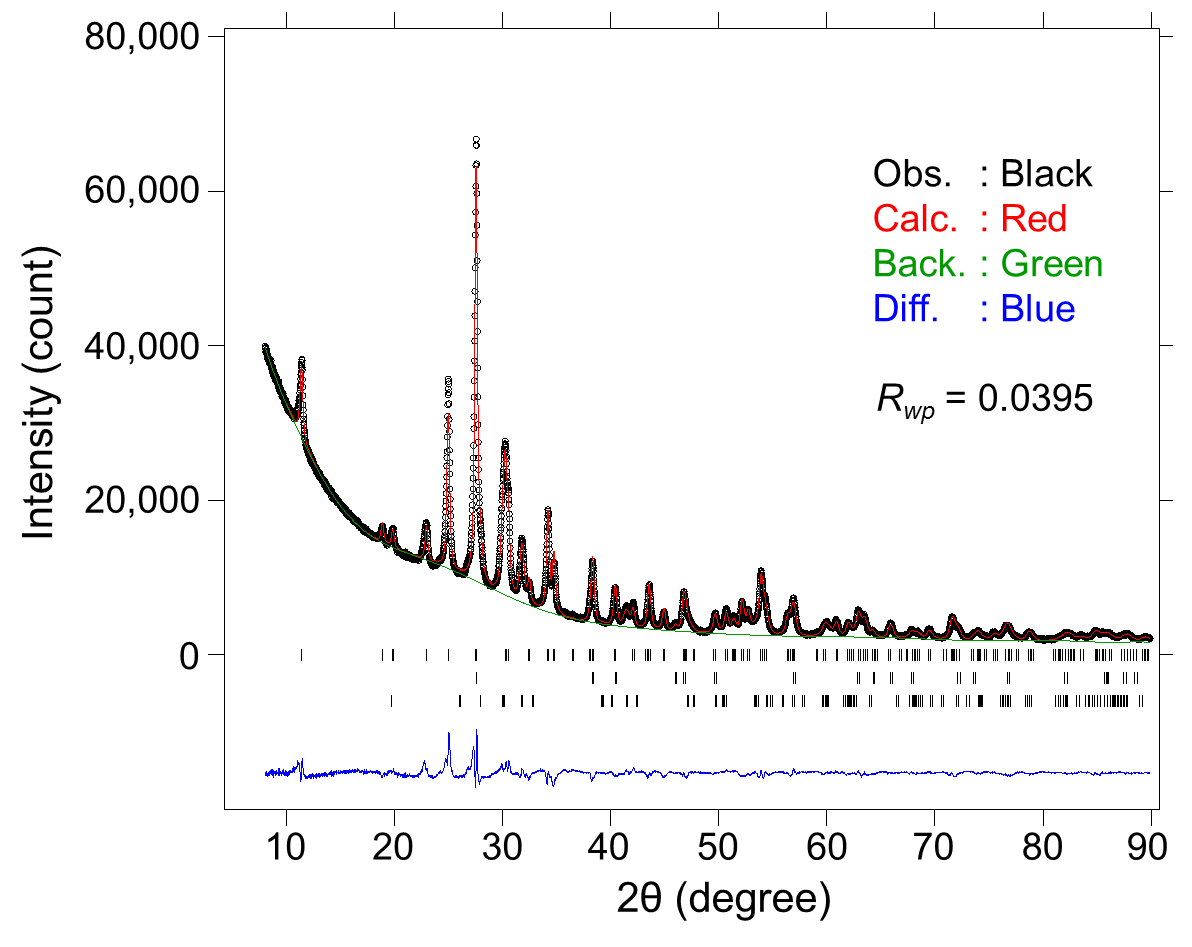


**Supplementary Figure 3** The Rietveld refinement fit of PXD for LiCa(AlH4)3 (*Rwp* = 0.0395). The observed, calculated background and difference between observed and calculated patterns are indicated circles, a red, green and blue lines, respectively. The positions of Bragg reflection are shown for LiCa(AlH4)3 (top), α-AlH3 (middle) and CaH2 (bottom). The refined weight fractions of LiCa(AlH4)3, α-AlH3 and CaH2 were 67 wt.%, 17 wt.% and 16 wt.%, respectively.


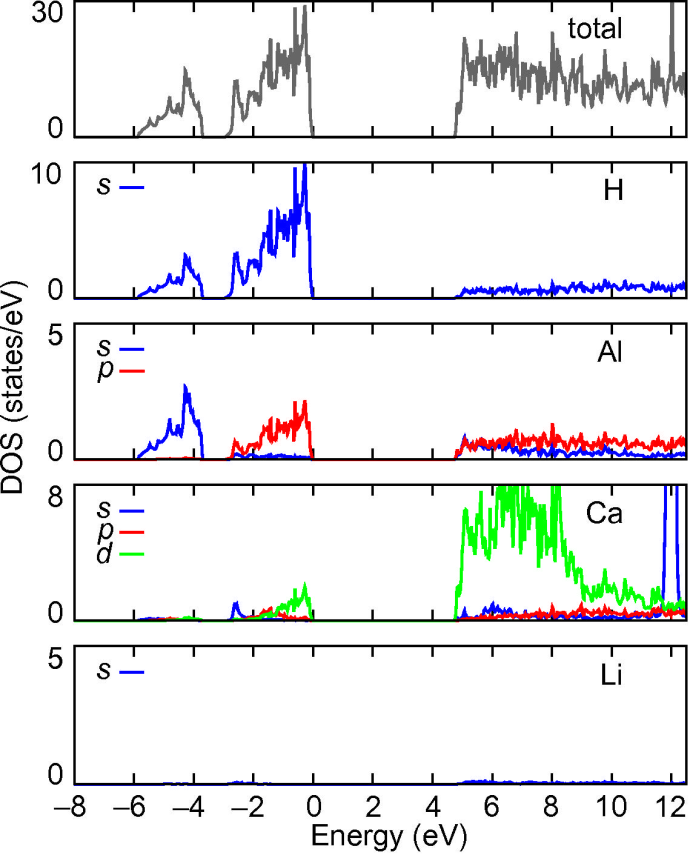


**Supplementary Figure 4** Total electronic DOS for the 34-atom unit cell of LiCa(AlH4)3 along with H *s*, Al *s*, Al *p*, Ca *s*, Ca *p*, Ca *d* and Li *s* projections. Zero energy is set at the valence-band maximum.


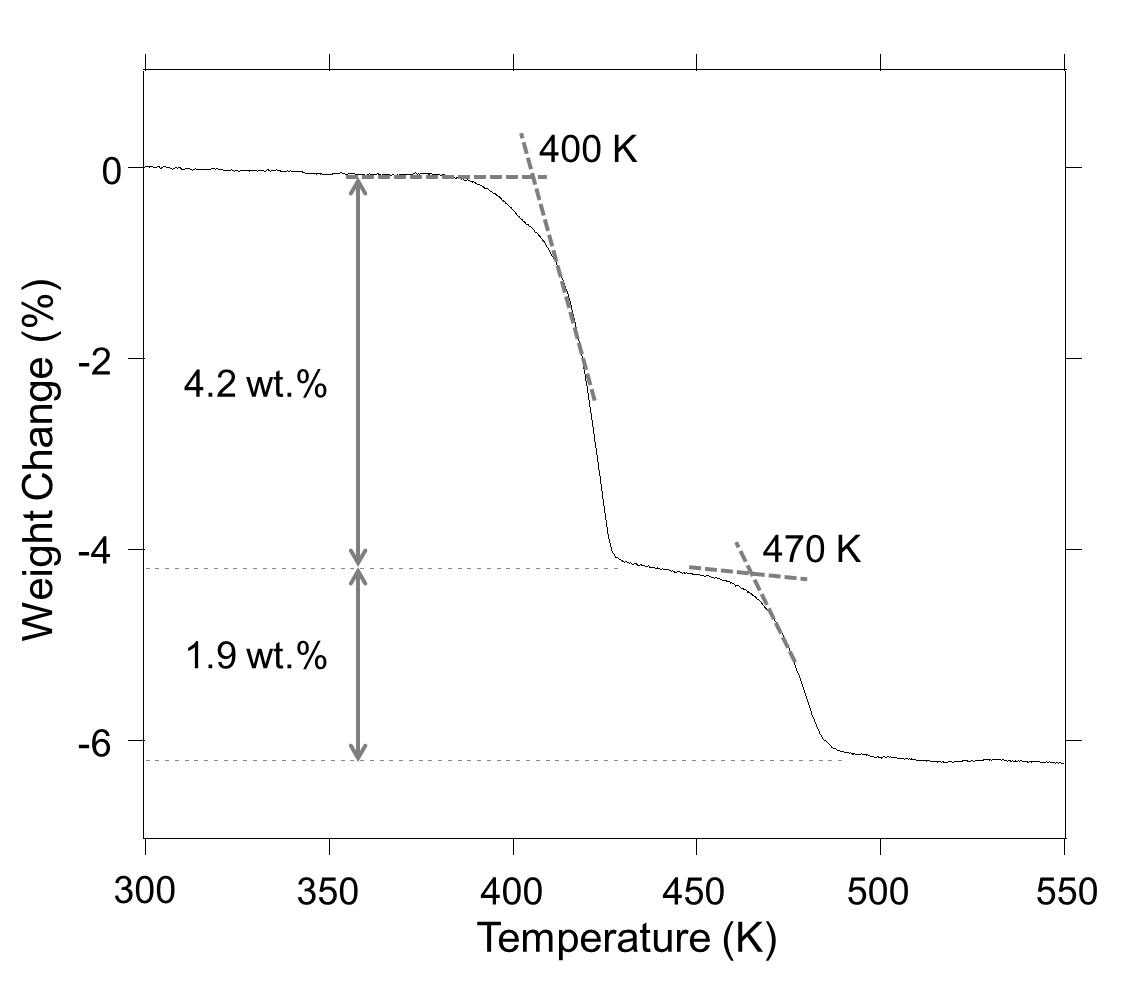


**Supplementary Figure 5** Thermogravimetric curve of LiCa(AlH4)3. The onset temperatures are 400 K and 470 K for the hydrogen release from LiCa(AlH4)3. The reactions at 400 K and 470 K are expected to be LiCa(AlH4)3 → LiCaAlH6 + 2Al + 3H2 (g) and LiCaAlH6 + 2Al → LiH + CaH2 + 3Al + 1.5H2 (g), respectively.


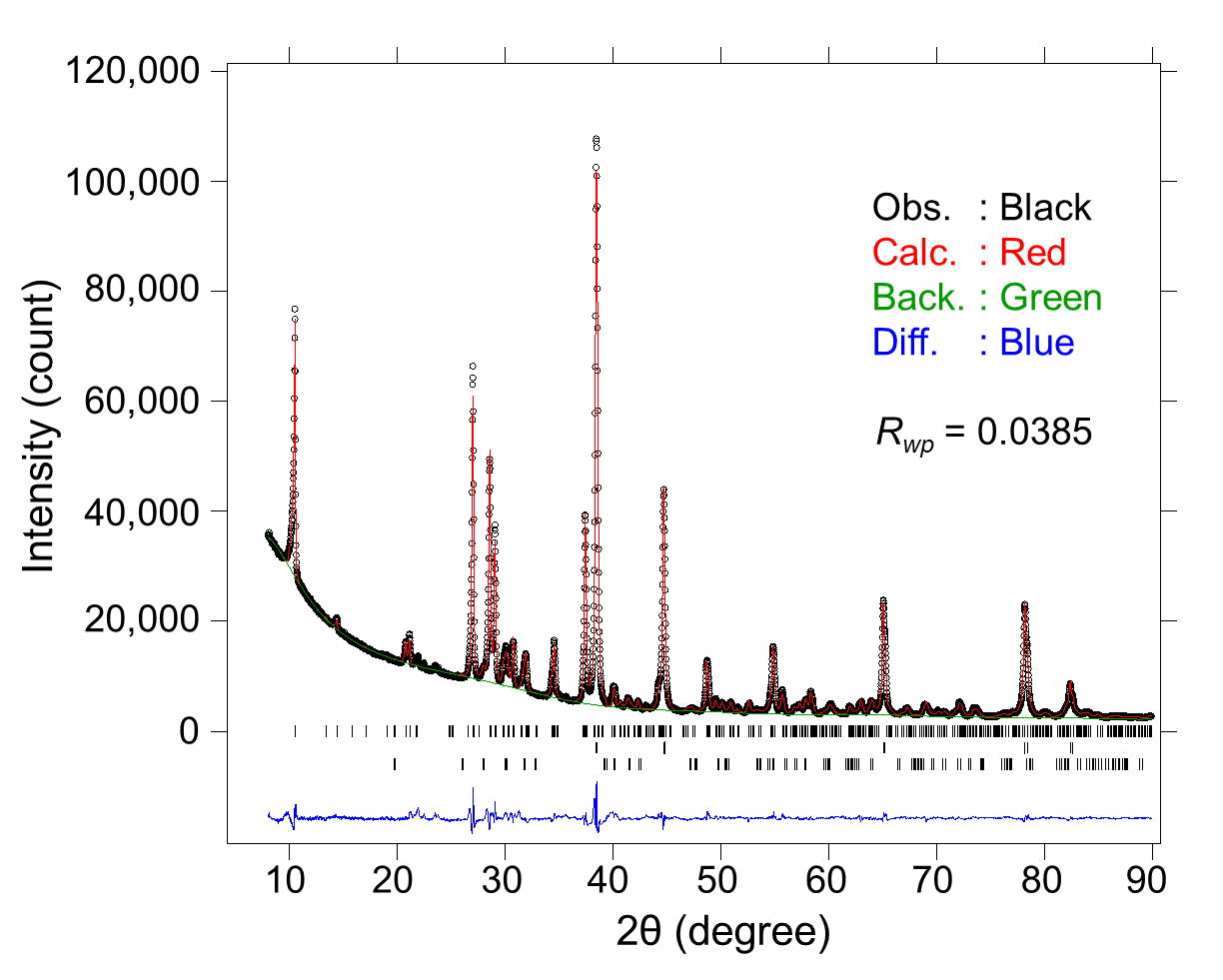


**Supplementary Figure 6** The Rietveld refinement fit of PXD for LiCaAlH6 (*Rwp* = 0.0385). The observed, calculated background and difference between observed and calculated patterns are indicated circles, a red, green and blue lines, respectively. The positions of Bragg reflection are shown for LiCaAlH6 (top), Al (middle) and CaH2 (bottom). The refined weight fractions of LiCaAlH6, Al and CaH2 were 47 wt.%, 48 wt.% and 5 wt.%, respectively.


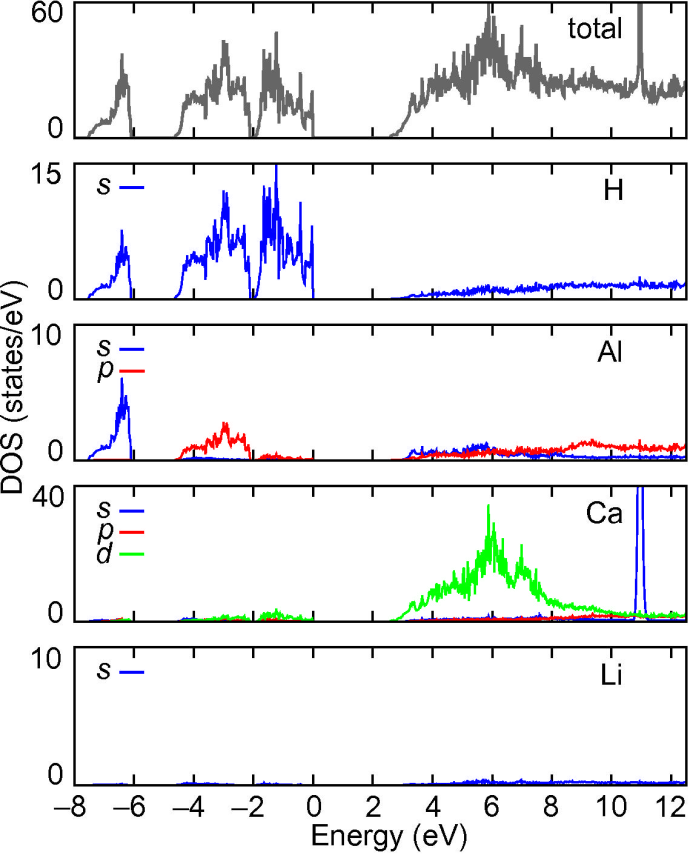


**Supplementary Figure 7** Total electronic DOS for the 72-atom unit cell of LiCaAlH6 along with H *s*, Al *s*, Al *p*, Ca *s*, Ca *p*, Ca *d* and Li *s* projections. Zero energy is set at the valence-band maximum.


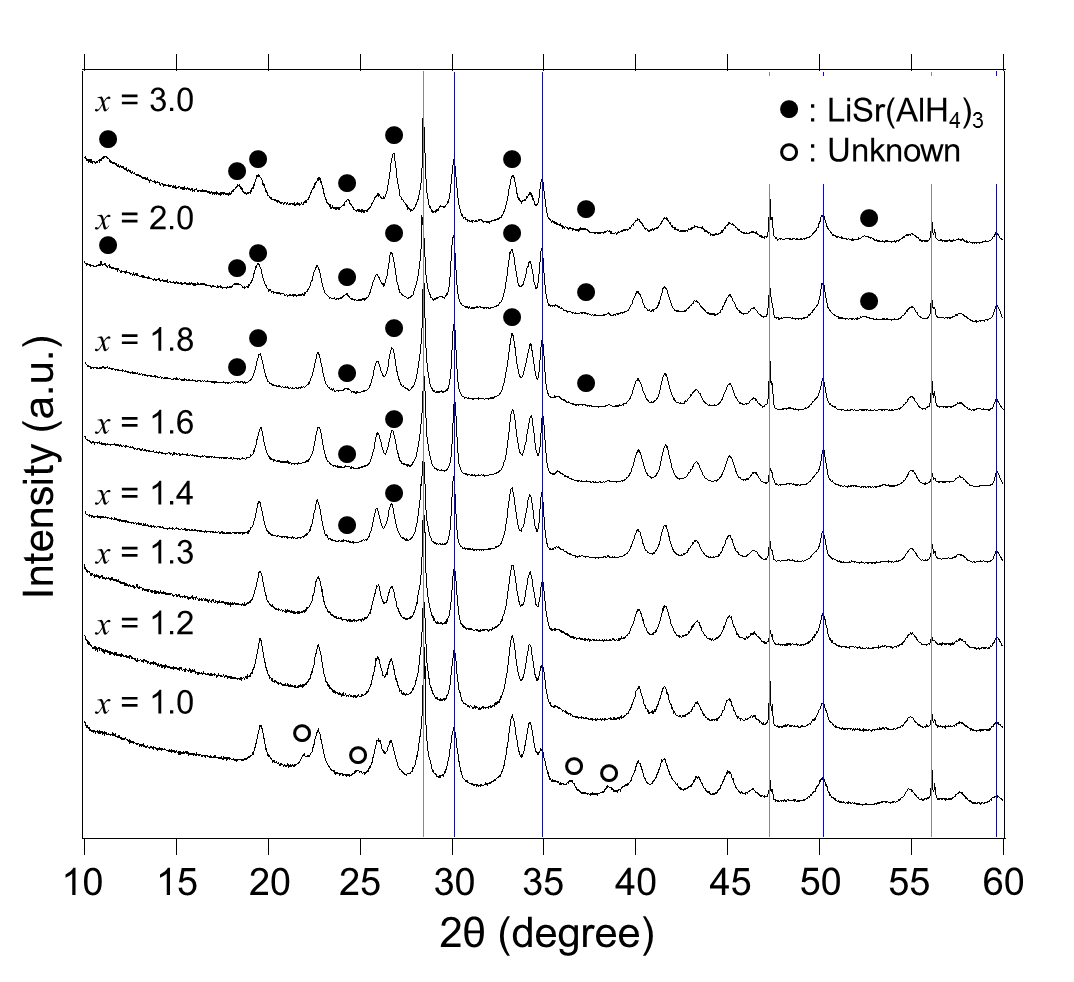


**Supplementary Figure 8** X-ray diffraction pattern of *x*LiAlH4­ + SrCl2 (*x* = 1.0 – 3.0).

Gray and blue lines shows peak positions of Si as an internal standard and LiCl, respectively. Peaks on 3.0LiAlH4 + SrCl2 except for LiCl and Sr(AlH4)Cl are indexed by a hexagonal unit cell with *a* = 9.150(2) Å and *c* = 6.074(5) Å which is slightly larger one than LiCa(AlH4)3 with a hexagonal unit cell (*a* = 8.9269 Å and *c* = 5.8941 Å). Therefore, the peaks are assigned by LiSr(AlH4)3.


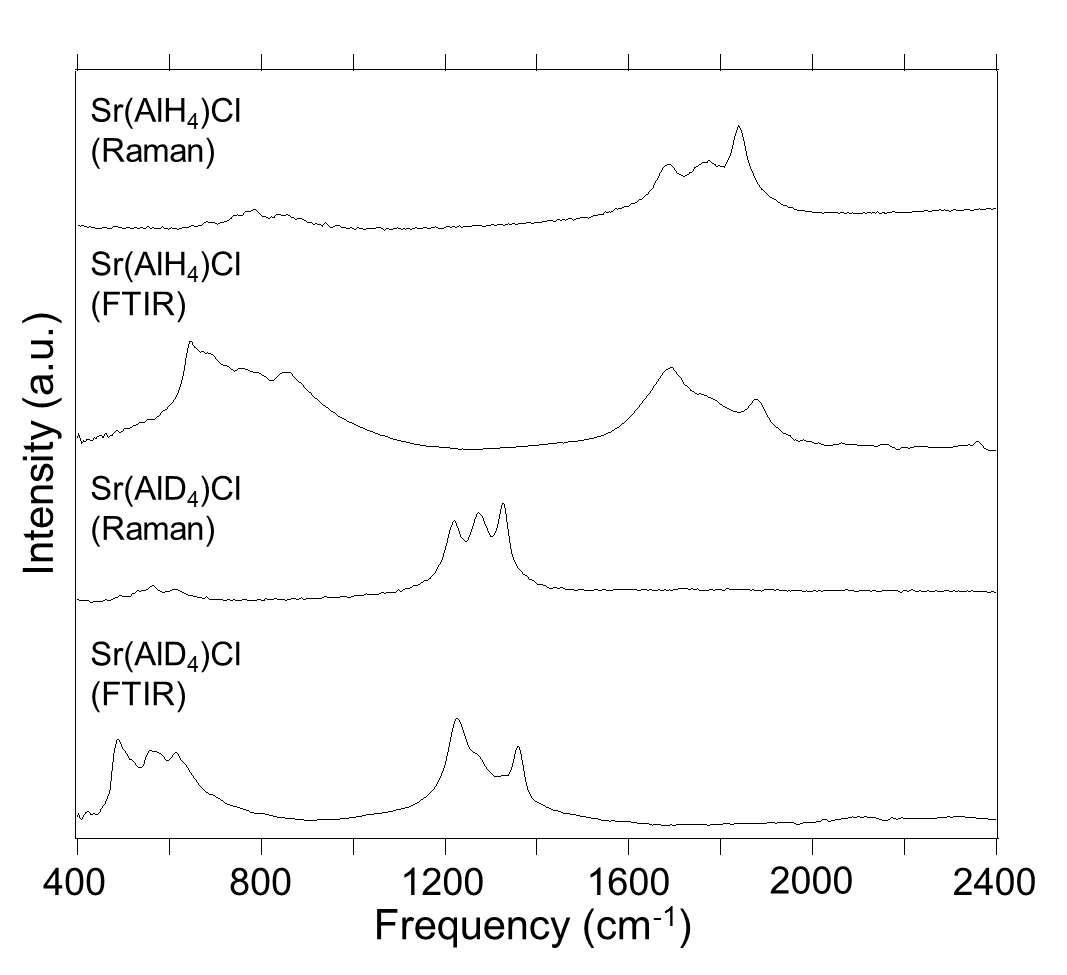


**Supplementary Figure 9** (top) Raman and (bottom) FTIR spectra of Sr(AlH4)Cl and Sr(AlD4)Cl. Compared with related tetra-alanate, NaAlH4,84 bending and stretching modes of [AlH4]– on Sr(AlH4)Cl are assigned at around 600-1200 and 1600-2000 cm–1, respectively.


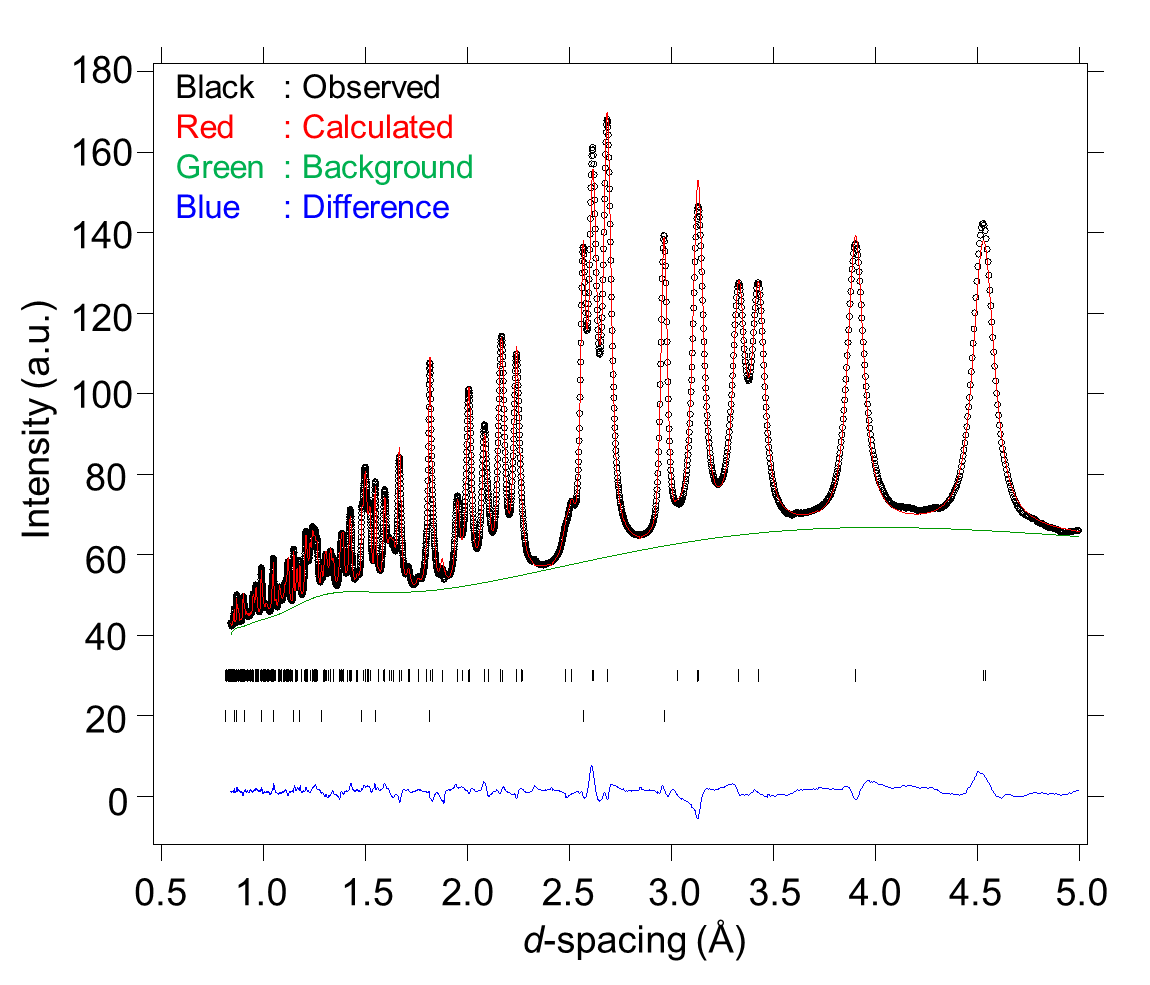


**Supplementary Figure 10** The Rietveld refinement fit of SR–PXD for Sr(AlD4)Cl (*Rwp* = 0.0135). The observed, calculated background and difference between observed and calculated patterns are indicated circles, a red, green and blue lines, respectively. The positions of Bragg reflection are shown for Sr(AlD4)Cl (top) and LiCl (bottom). The refined weight fractions of Sr(AlD4)Cl and LiCl were 78 wt.% and 22 wt.%, respectively.


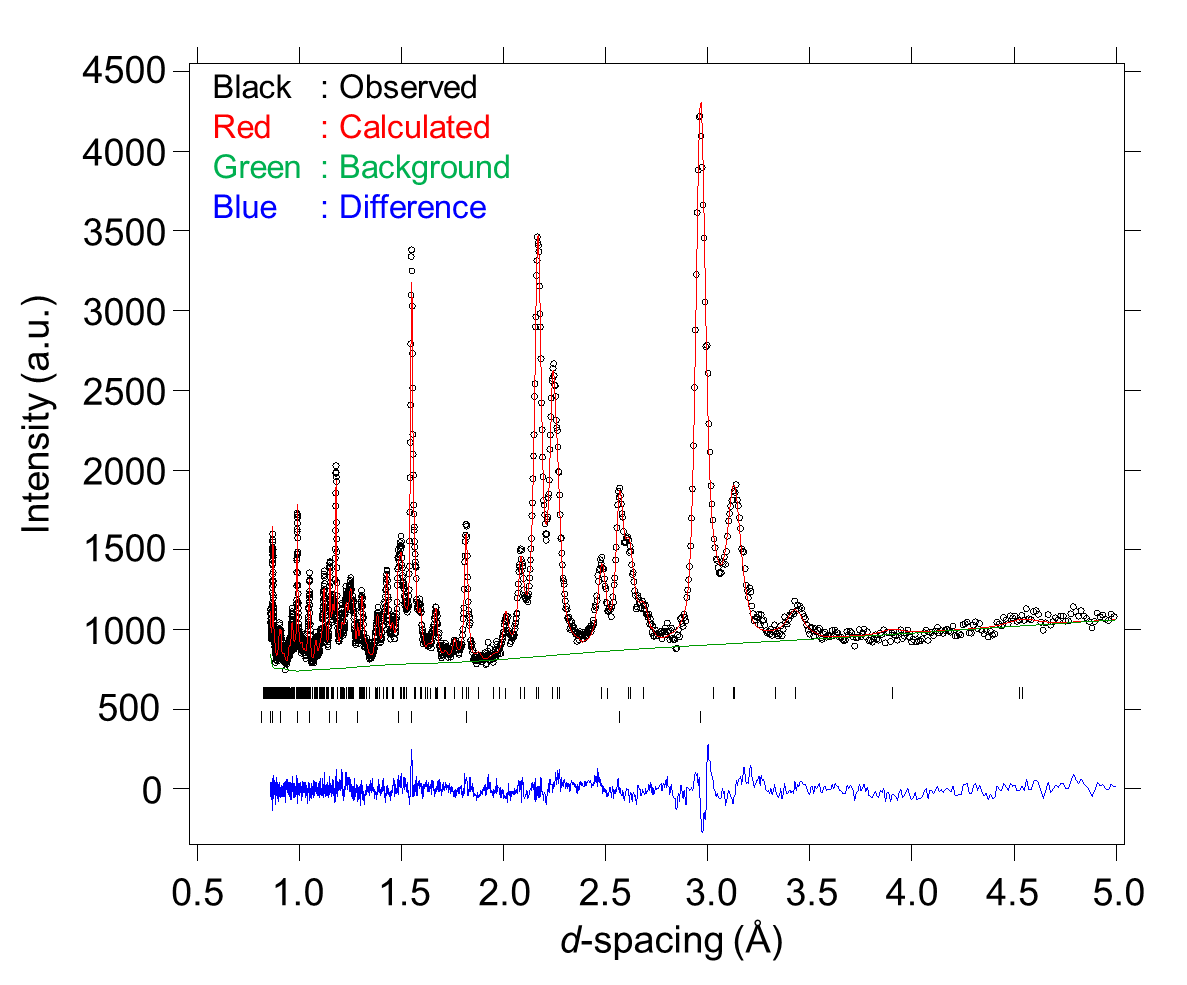


**Supplementary Figure 11** The Rietveld refinement fit of PND for Sr(AlD4)Cl (*Rwp* = 0.0330). The observed, calculated background and difference between observed and calculated patterns are indicated circles, a red, green and blue lines, respectively. The positions of Bragg reflection are shown for Sr(AlD4)Cl (top) and LiCl (bottom). The refined weight fractions of Sr(AlD4)Cl and LiCl were 76 wt.% and 24 wt.%, respectively.

**Estimation of thermodynamical radii of complex anion**

Most of the ionic compounds are located around *V*unit/*Z* = 1.45*V*ion. This indicates that thermochemical radii of complex anions can be obtained from *V*unit/*Z* = 1.45*V*ion. Therefore, we estimated thermochemical radii of complex anions [CrH7]5–, [MnH6]5–, [FeH6]4–, [CoH5]4–, [NiH4]4–, [CuH­4]3–, [ZnH4]2–, [B3H8]–, [B10H10]2–, [B12H12]2–, 85–93 which have not been reported. The estimated thermochemical radii and IFFs are compared with ones from a conventional method (equation 1) and listed in Supplementary Table 19.

In the conventional method, it is difficulty in estimation of lattice energy of a crystal except for typical crystal structure type such as NaCl–type. Then, lattice energies are estimated using an approximate equation which has been reported Jenkins et al.94

Although the approximant equation provides lattice energies for untypical crystal structure types, it is not available for ionic compounds with multi–cations and anions. The IFFs show relatively larger. In particular, IFFs of Li2B12H12 and Na2B12H12 are over IFF = 1.0. However, there is no limitation of number of ions and IFFs are less than 1.0 when we used the IFF plot. In order to consider validity of the thermochemical radii obtained from the conventional method and the IFF plot, we compare the thermochemical radius of [NiH4]4– with a similar tetrahedral complex [AlH4]– anion, of which thermochemical radius has been reported as 2.26 Å.4 The thermochemical radii using the conventional method and IFF plot are 2.35 Å and 2.22 Å, respectively. Focused on the distances between center and ligand atoms in [NiH4]4– and [AlH4]–, they are 1.52 – 1.57 Å85 and 1.60 – 1.65 Å,80 respectively. Therefore, the thermochemical radius of [NiH4]4– is ought to be smaller than [AlH4]–. This indicates that IFF plot provides more reasonable thermochemical radii than the conventional method.

**Supplementary Table 21** Thermochemical radii of complex anions and their IFFs obtained from IFF plot and the conventional method. In the conventional method, the thermochemical radii in Mg3CrH8 (Mg2+, [CrH7]5– and H–), Mg3MnH7 (Mg2+, [MnH6]5– and H–) and Ba7Cu3H17 (Ba2+, [CuH4]3– and H–)) cannot be estimated.

|  | Thermodynamical radii (Å) | | IFF | | |
| --- | --- | --- | --- | --- | --- |
|  | IFF plot | Conventional | Hydrides | IFF plot | Conventional |
| [CrH7]5– | 2.37 | – | Mg3CrH8 | 0.70 | – |
| [MnH6]5– | 2.34 | – | Mg3MnH7 | 0.69 | – |
| [FeH6]4– | 2.26 | 2.46 | Mg2FeH6  Ca2FeH6 | 0.75  0.65 | 0.96  0.81 |
| [CoH5]4– | 2.19 | 2.31 | Mg2CoH5 | 0.69 | 0.81 |
| [NiH4]4– | 2.22 | 2.35 | Mg2NiH4 | 0.69 | 0.82 |
| [CuH4]3– | 2.41 | – | Ba7Cu3H17 | 0.69 | – |
| [ZnH4]2– | 2.46 | 2.75 | K2ZnH4  Rb2ZnH4  Cs2ZnH4 | 0.72  0.69  0.67 | 0.94  0.88  0.83 |
| [B3H8]– | 2.62 | 2.52 | NaB3H8  CsB3H8 | 0.73  0.66 | 0.65  0.61 |
| [B10H10]2– | 3.29 | 3.50 | Na2B10H10  K2B10H10  Rb2B10H10 | 0.71  0.70  0.68 | 0.84  0.82  0.79 |
| [B12H12]2– | 3.52 | 3.96 | Li2B12H12  Na2B12H12  K2B12H12  Rb2B12H12  Cs2B12H12 | 0.84  0.72  0.68  0.66  0.62 | 1.19  1.02  0.94  0.90  0.83 |

**Bonding nature of Al – H in AlH3**

AlH3, which has the ability to be used in hydrogen storage due to the high gravimetric hydrogen density (10.1 wt.%), has been reported to from four modifications (α, α’, β and γ–AlH3).80 Assuming ionic radii of Al3+ as 0.535 Å and H– as 1.40 Å, the IFFs are estimated (Supplementary Table 20) as well as the binary hydrides. The IFF of α–AlH3 shows 1.05. Even though β–AlH3 has channels in the crystal structure, it is 0.77 which means a denser ionic compound than averaged one (0.69). This originates from chemical bonding between Al and H in AlH3 because the bonding nature between Al and H has been reported to contain a covalency. 95, 96

As mentioned above, ionic radius can be estimated from IFF plot (*V*unit/*Z* = 1.45*V*ion). Assuming ionic radius of Al3+ as 0.535 Å, ionic radius of H– is estimated be 1.28 Å and the IFFs of α, α’, β and γ–AlH3 are reasonable (Supplementary Table 18). The slightly smaller ionic radius of H– than the reported one (1.40 Å) suggests that bonding nature between Al and H could not be an ideal ionic bonding but containing of covalent one as reported ref. 95 and 96.

**Supplementary Table 22** IFFs of α, α’, β and γ–AlH3 obtained from reported and reestimated ionic radius of H–

|  | IFF | |
| --- | --- | --- |
|  | Radius of H– (1.40 Å) | Radius of H– (1.28 Å) |
| α–AlH3 | 1.05 | 0.81 |
| α’–AH3 | 0.89 | 0.69 |
| β–AlH3 | 0.77 | 0.59 |
| γ–AlH3 | 0.93 | 0.71 |

**5. Supplementary References**

1. Kapustinskii, A. F. Lattice energy of ionic crystal. *Q. Rev. Chem. Soc.* **10**, 283 – 294 (1956).

2. Jenkins, H. D. B. & Thakur, K. P. Reappraisal of thermochemical radii for complex ions. *J. Chem. Educ.* **56**, 576 – 577 (1979).

3. Glasser, L. Lattice energies of crystals with multiple ions: A generalized Kapustinskii equation. *Inorg. Chem.* **34**, 4935 – 4936 (1995).

4. D. R. Lide, CRC Handbook of Chemistry and Physics 88th edition, CRC Press, 2007.

5. Taylor, D. Thermal expansion data: II. Binary oxides with the fluorite and rutile structure, MO2, and the antifluorite structure M2O. *Trans. Br. Ceram. Soc.* **83**, 32 – 37 (1984).

6. Smith, D.K. & Leider, H.R. Low-temperature thermal expansion of LiH, MgO and CaO. *J. Appl. Crystallor.* **1**, 246 – 249 (1968).

7. Cooper, M.J. The analysis of powder diffraction data. *Acta Crystallogr. A* **38**, 264 –269 (1982).

8. Cromer, D.T. & Herrington, K. The structures of anatase and rutile. *J. Am. Chem. Soc.* **77**, 4708 – 4709 (1955).

9. Bacon, G.E. A neutron diffraction study of magnesium aluminium oxide. *Acta Crystallogr.* **5**, 684 – 686 (1952).

10. Post, J. E. & Heaney, P. J. Neutron and synchrotron X-ray diffraction study of the structures and dehydration behaviors of ramsdellite and "groutellite". *Am. Mineral.* **89**, 969 – 975 (2004).

11. Sawada, H. Electron density study of garnets: *Z*3Al2Si3O12 (*Z* = Mg, Fe, Mn, Ca) and Ca3Fe2Si3O­12. *J. Solid State Chem.* **142**, 273 – 278 (1999).

12. Tejada–Rosales, E. M., Rodriguez–Carvajal, J., Casan–Pastor, N., Alemany, P., Ruiz, E., El–Fallah, M. S., Alvarez, S. & Gomez–Romero, P. Room–temperature synthesis and crystal, magnetic and electronic structure of the first silver copper oxide. Inorg. Chem. **41**, 6604 – 6613 (2002).

13. Peel, M. D., Thompson, S. P., Daoud-Aladine, A., Ashbrook, S. E. & Lightfoot, P. New twists on the perovskite theme: crystal structures of the elusive phases R and S of NaNbO3. *Inorg. Chem.* **51**, 6876 – 6889 (2012).

14. Ahtee, M. & Darlington, C. N. W. Structures of NaTaO3 by neutron powder diffraction. *Acta Crystallogr. B* **36**, 1007 – 1014 (1980).

15. Katz, L. & Megaw, H. D. The structure of potassium niobate at room temperature: The solution of a pseudosymmetric structure by Fourier methods. *Acta Crystallogr.* **22**, 639 – 648 (1967).

16. Zhurova, E. A., Ivanov, Y., Zavodnik, V. & Tsirelson, V. G. Electron density and atomic displacements in KTaO­3, *Acta Crystallogr. B* **56**, 594 – 600 (2000).

17. Sasaki, S., Prewitt, C. T. & Bass, J. D. Orthorhombic perovskite CaTiO3 and CdTiO3: structure and space group. *Acta Crystallogr. C* **43**, 1668 – 1674 (1987).

18. Kobayashi, H., Nagata, M., Kanno, R. & Kawamoto, Y. Structural characterization of the orthorhombic perovskites: (*A*RuO3 (*A* = Ca, Sr, La, Pr)). *Mater. Res. Bull.* **29**, 1271 – 1280 (1994).

19. Brous, J., Fankuchen, I. & Banks, E. Rare earth titanates with a perovskite structure. *Acta Crystallogr.* **6**, 67 – 70 (1953).

20. Bushmeleva, S. N., Pomjakushin, V. Yu., Pomjakushina, E. V., Sheptyakov, D. V. & Balagurov, A. M. Evidence for the band ferromagnetism in SrRuO3 from neutron diffraction. *J. Magn. Magn. Mater.* **305**, 491 – 496 (2006).

21. Megaw, H.D. Crystal structure of barium titanate. *Nature* **155**, 484 – 485 (1945).

22. Levin, I. *et al.* Phase equilibria, crystal structures and dielectric anomaly in the BaZrO3 – CaZrO3 system. *J. Solid State Chem.* **175**, 170 – 181 (2003).

23. Cheng, J. G. *et al.* High–pressure synthesis of the BaIrO3 perovskite. A Pauli paramagnetic metal with a Fermi liquid ground state. *Phys. Rev. B* **88**, 205114 (2013).

24. Malavasi, L., Kim Hyun–Jeong & Proffen, T. Local and average structures of the proton conducting Y-doped BaCeO3 from neutron diffraction and neutron pair distribution function analysis. *J. Appl. Phys.* **105**, 123519 (2009).

25. Kuroiwa, Y. *et al.* Evidence for Pb-O covalency in tetragonal PbTiO3. *Phys. Rev. Lett.* **87**, 217601 (2001).

26. Teslic, S. & Egami, T. Atomic structure of PbZrO3 determined by pulsed neutron diffraction. Acta Crystallogr. B 54, 750 – 765 (1998).

27. Fujishita, H. *et al.* A study of structures and order parameters in antiferroelectric PbHfO3 by synchrotron radiation. *J. Phys. Soc. Jpn.* **74**, 2743 – 2747 (2005).

28. Belik, A. A. *et al.* BiScO3: Centrosymmetric BiMnO3–type oxide. *J. Am. Chem. Soc.* **128**, 706 – 707 (2006).

29. Chen, W. –T. *et al.* Robust antiferromagnetism and structural disorder in BixCa1-xFeO3 perovskites. *Chem. Mater.* **21**, 2085 – 2093 (2009).

30. Eitel, M. & Greedan, J. E. A high resolution neutron diffraction study of the perovskite LaTiO3. *J. Less–Comm. Mater.* **116**, 95 – 104 (1986).

31. Garcia–Munoz, J. L., Rodriguez–Carvajal, J., Lacorre, P. & Torrance, J. B. Neutron-diffraction study of *R*NiO3 (*R* = La, Pr, Nd, Sm): Electronically induced structural changes across the metal-insulator transition. *Phys. Rev. B.* **46**, 4414 – 4425 (1992).

32. Sirdeshmukh, D. B., Sirdeshmukh, L. & Subhadra, K. G., Alkali halides: A handbook of physical properties. Springer–Verlag, Berlin, 2001.

33. Vidal-Valat, G., Vidal, J. –P., Zeyen, C. M. E. & Kurki–Suonio, K. *Acta Crystallogr. B* **35**, 1584 – 1590 (1979).

34. Batchelder, D.N. & Simmons, R.O. Lattice constants and thermal expansivities of silicon and of calcium fluoride between 6 and 322 K. *J. Chem. Phys.* **41**, 2334 – 2329 (1964).

35. Forxyth, J. B., Wilson, C. C. & Sabine, T. M. A time-of-flight neutron diffraction study of anharmonic thermal vibrations in SrF2, at The spallation neutron source ISIS, *Acta Crystallogr. A* **45**, 244 – 247 (1989).

36. Radtke, A. S. & Brown, G.E. Frankdicksonite, BaF2, a new mineral from Nevada, *Am. Mineral.* **59**, 885 – 888 (1974).

37. Partin, D.E. & O’Keefe, M. The structure and crystal chemistry of magnesium chloride and cadmium chloride. *J. Solid State Chem.* **95**, 176 – 183 (1991).

38. van Bever, A. K. & Nieuwenkamp, W. Die kristallstruktur von calciumchlorid, CaCl2. *Z. Kristallogr.* **90**, 374 – 376 (1935).

39. Ott, H. Die strukturen von MnO, MnS, AgF, NiS, SnI4, SrCl2, BaF2, praezisionsmessungen einiger alkalihalogenide. *Z. Kristallogr.* **63**, 222 – 230 (1926).

40. Brackett, E. B., Brackett, T. E. & Sass, R.L. The crystal structure of barium chloride, barium bromide, and barium iodide. *J. Phys. Chem.* **67**, 2132 – 2135 (1963).

41. Ferrari, A. & Giorgi, F. Crystal structure of the bromides of divalent metals. *Atti Accad. Naz. Lincei.* **9**, 782 – 789 (1929).

42. Brackett, E. B., Brackett, T. E. & Sass, R. L. The crystal structure of calcium bromide. *J. Inorg. Nucl. Chem.* **25**, 1295 – 1296 (1963).

43. Sass, R. L., Brackett, T. E. & Brackett, E. B. The crystal structure of strontium bromide. *J. Phys. Chem.* **67**, 2862 – 2863 (1963).

44. Blum, H. Die kristallstruktur des wasserfreien magnesiumjodids und calciumjodids. *Z. Phys. Chem. B* **22**, 298 – 304 (1933).

45. Baernighausen, H., Beck, H., Grueninger, H. W., Rietschel, E. T. & Schultz, N. Neue (AB2) – strukturtypen mit siebenfach koordiniertem kation. *Z. Kristallogr.* **128**, 430 (1969).

46. Vidal, J. P. & Vidal–Valat, G. Accurate Debeye-Waller factors of 7LiH and 7LiD by neutron diffraction. *Acta Crystallogr. B* **42**, 131 – 137 (1986).

47. Shull, C. G., Wollan, E. O., Morton, G. A. & Davidson, W. L. Neutron diffraction studies of NaH and NaD. *Phys. Rev.* **73**, 842 – 847 (1948).

48. Kohlmann, H., Zho Y., Nicol, M. F. & McClure, J. Crystal structure of alpha-MgD2 under high pressures by neutron powder diffraction. *Z. Kristallogr.* **223**, 706 – 710 (2008).

49. Wu, H., Zhou, W., Udovic, T. J., Rush, J. J. & Yildirim, T. Structure and vibrational spectra of calcium hydride and deuteride. J. Alloys Compd. **436**, 51 – 55 (2007).

50. Brese, N. E., O'Keeffe, M. & von Dreele, R. B. Synthesis and crystal structure of SrD2 and SrND and bond valence parameters for hydrides. *J. Solid State Chem.* **88**, 571 – 576 (1990).

51. Ikeda, K., Sato, T. & Orimo, S. Perovskite–type hydrides – synthesis, structures and properties. *Int. J. Mat. Res.* **99**, 471 – 478 (2008).

52. Hartman, M. R., Rush, J. J., Udvic, T. J., Bowman Jr, R. C. & Hwang, S. –J. Structure and vibratinal dynamics of LiBD4. *J. Solid State Chem.* **180**, 1298 – 1305 (2007).

53. Fischer, P. & Züttel, A. Order–disorder phase transition in NaBD4. *Mater. Sci. Form* **443 – 444**, 287 – 290 (2004).

54. Renaudin, G., Gomes, S., Hagemann, H., Keller, L. & Yvon, K. Structural and spectroscopic studies on the alkali borohydrides *M*BH4 (*M* = Na, K, Rb, Cs). *J. Alloys Compd.* **375**, 98 – 106 (2004).

55. Černý, R., Filinchuk, Y., Hagemann, H. & Yvon, K. Magnesium borohydride: synthesis and crystal structure. *Angew. Chem. Int. Ed.* **46**, 5765 – 5767 (2007).

56. Her, J. –H. *et al.* Structure of unsolvated magnesium borohydride Mg(BH4)2. *Acta Crystallogr. B* **63**, 561 – 568 (2007).

57. Filinchuk, Y. *et al.* Porous and dense magnesium borohydride frameworks: synthesis, stability, and reversible absorption of guest species. *Angew. Chem. Int. Ed.* **50**, 11162 – 11166 (2011).

58. Buchter, F. *et al.* Structure of Ca(BD4)2 β–phase from combined neutron and synchrotron x-ray diffraction data and density functional calculations. *J. Phys. Chem. B* **112**, 8042 – 8048 (2008).

59. Buchter, F. et al. Structure of the orthorhombic γ–phase and phase transitions of Ca(BD4)­. *J. Phys. Chem. C* **113**, 17223 – 172230 (2009).

60. Ravnsbæk, D. B. *et al.* Novel alkali earth borohydride Sr(BH4)2 and borohydride–chloride Sr(BH4)Cl. *Inorg. Chem.* **52**, 10877 – 10885 (2013).

61. Černý, R., Penin, N., Hagemenn, H. & Filinchuk, Y. The first crystallographic and spectroscopic characterization of a 3*d*-metal borohydride: Mn(BH4)2. *J. Phys. Chem. C* **113**, 9003 – 9007 (2009).

62. Sato, T. *et al.* Experimental and computational studies on solvent–free rare–earth metal borohydrides *R*(BH4)3 (*R* = Y, Dy, and Gd). *Phys. Rev. B* **77**, 104114 (2008).

63. Aldridge, S. *et al.* Some tetrahydroborate derivatives of aluminium: crystal structures of demethylaluminium tetrahydroborate and the α and β phases of aluminium tris(tetrahydroboate) at low temperature. *J. Chem. Soc. Dalton Trans.* 1007 – 1012 (1997).

64. Bird, P. H. & Churchill, M. R. The crystal structure of zirconium(IV) borohydride (at –160º). *Chem. Commun. (London)* **8**, 403 (1967).

65. Broach, R. W., Chuang, I. –S., Marks, T. J. & Williams, J. M. Metal characterization of tridentate tetrahydroborate ligation to a transition–metal ion. Structure and bonding in Hf(BH4)4 by single–crystal neutron diffraction. *Inorg. Chem.* **22**, 1081 (1983).

66. Ravnsbæk, D. B., Sørensen, L. H., Filinchuk, Y., Besenbacher, F. & Jensen, T. R. Screening of metal borohydrides by mechanochemistry and diffraction. *Angew. Chem. Int. Ed.* **51**, 3582 – 3586 (2012).

67. Schouwink, P. *et al.* Bimetallic borohydrides in the system *M*(BH­4)2–KBH4 (*M* = Mg, Mn): On the structural diversity. *J. Phys. Chem. C* **116**, 10829 – 10840 (2012).

68. Černý, R. *et al.* Structure and characterization of KSc(BH4)4. *J. Phys. Chem. C* **114**, 19540 – 19549 (2010).

69. Jaroń, T. & Grochala, W. Probing Lewis acidity of Y(BH4)3 via its reactions with MBH4 (M= Li, Na, K, NMe4). *Dalton Trans.* **40**, 12808 – 12817 (2011).

70. Černý, R. *et al.* Potassium zinc borohydrides containing triangular [Zn(BH4)3]– and tetrahedral [Zn(BH4)xCl4–x]2– anions. J. Phys. Chem. C **116**, 1563 – 1571 (2012).

71. Nickels, E. A. *et al.* Tuning the decomposition temperature in complex hydrides: Synthesis of a mixed alkali metal borohydride. *Angew. Chem. Int. Ed.* **47**, 2817 – 2819 (2008).

72. Hagemann, H. *et al.* LiSc(BH4)4: A novel salt of Li+ and discrete Sc(BH4)4– complex anions. *J. Phys. Chem. A* **112**, 7551 – 7555 (2008).

73. Černý, R. *et al.* AZn2(BH4)5 (A= Li, Na) and NaZn(BH4)3: Structural studies. *J. Phys. Chem. C* **114**, 19127 – 19133 (2010).

74. Seballos, L., Zhang, J. Z., Rönnebro, E., Herberg, J. L. & Majzoub, E. H. Metastability and crystal structure of the bialkali complex metal borohydride NaK(BH4)2. *J. Alloys. Compd.* **476**, 446 – 450 (2009).

75. Černý, R. *et al.* NaSc(BH4)4: A novel scandium–based borohydride. *J. Phys. Chem. C* **114**, 1357 – 1364 (2010).

76. Balogh, M. P., Jones, C. Y., Herbst, J. F., Hector, L. G. Jr. & Kundrat, M. Crystal structures and phase transformation of deuterated lithium imide. *J. Alloys Compd.* **420**, 326 – 336 (2006).

77. Dolci, F. *et al.* Magnesium imide: synthesis and structure determination of an unconventional alkaline earth imide from decomposition of magnesium amide. *Inorg. Chem.* **50**, 1116 – 1122 (2011).

78. Sørby, M. H. *et al.* The crystal structure of LiND2 and Mg(ND2)2. *J. Alloys Compd.* **428**, 297 – 301 (2007).

79. Wu, H., Zhou, W., Udovic, T. J., Rush, J. J. & Yildirim, T. Structure and crystal chemistry of Li2BNH6 and Li4BN3H10. *Chem. Mater.* **20**, 1245 – 1247 (2008).

80. Hauback, B.C. Structures of aluminium–based light weight hydrides. *Z. Kristallogr.* **223**, 636 – 648 (2008).

81. Sato, T. *et al.* Syntheses, Crystal structures, and thermal analyses of solvent–free Ca(AlD4)2 and CaAlD5. *J. Alloys Compd.* **487**, 472 – 478 (2009).

82. Pommerin, A., Wosylus, A., Felderhoff, M., Schüth, F. & Weidenthaler, C. Synthesis, Crystal Structures, and Hydrogen-Storage Properties of Eu(AlH4)2 and Sr(AlH4)2 and of Their Decomposition Intermediates, EuAlH5 and SrAlH5. *Inorg. Chem.* **51**, 4143 – 4150 (2012).

83. Weidenthaler, C. *et al.* Complex rare–earth aluminum hydrides: Mechanochemical preparation, crystal structure and potential for hydrogen storage. *J. Am. Chem. Soc.* **131**, 16735 – 16743 (2009).

84. Majzoub, E. M., McCarty, K. F. & Ozoliņš, V. Lattice dynamics of NaAlH4 from high–temperature single–crystal Raman scattering and ab initio calculations: Evidence of highly stable [AlH4]− anions. *Phys. Rev. B* **71**, 024118 (2005).

85. Yvon, K. Complex transition–metal hydrides. *Chimia* **52**, 613 – 619 (1998).

86. Takagi, S. *et al.* True Boundary for the Formation of Homoleptic Transition–Metal Hydride Complexes. *Angew. Chem. Int. Ed.* **54**, 5650 – 5653 (2015).

87. Huang Z. *et al.* A simple and efficient way to synthesize unsolvated sodium octahydrotriborate. *Inorg. Chem.* **49**, 8185 – 8187 (2010).

88. Deiseroth, H. J., Sommer, O., Binder, H., Wolfer, K. & Frei, B. CsB3H8: Kristallstruktur und Optimierung der Synthese. *Z. Anorg. Allg. Chem.* **571**, 21 – 28 (1989).

89. Hofmann, K. & Albert, B. Crystal structures of M2[B10H10] (M = Na, K, Rb) via real space simulated annealing powder techniques. *Z. Kristallogr.* **220**, 142 – 146 (2005).

90. Her J. –H. *et al.* Crystal structure of Li2B12H12: A possible intermediate species in the decomposition of LiBH4. *Inorg. Chem.* **47**, 9757 – 9759 (2008).

91. Her J. –H., Zhou, W., Stavila, V., Brown, C. M. & Udovic, T. J. Role of cation size on the structural behavior of the alkali-metal dodecahydro-*closo*-dodecaborates. *J. Phys. Chem. Lett.* **113**, 11187 – 11189 (2009).

92. Tiritiris, I. & Schleid, T. Die dodekahydro-*closo*-dodekaborate M2[B12H12] der schweren Alkalimetalle (M+ = K+, Rb+, NH4+, Cs+) und ihre formalen iodid-addukte M3I[B12H12] (≡ [MI]∙M2[B12H12]). *Z. Anorg. Allg. Chem.* **629**, 1390 – 1402 (2003).

93. Tiritiris, I., Schleid, T., Mueller, K. & Preetz, W. Strukturelle Untersuchungen an Cs2[B12H12]. *Z. Anorg. Allg. Chem.* **626**, 323 – 325 (2000).

94. B. Jenkins, H. D. B., Roobottom, H. K., Passmore, J. & Glasser, L. Relationships amon ionic lattice energies, molecular (formula unit) volumes, and thermochemical radii. *Inorg. Chem.* **38**, 3609 – 3620 (1999).

95. Takeda, Y. *et al.* Electronic structure of aluminium trihydride studied using soft x–ray emission and absorption spectroscopy. *Phys. Rev. B* **84**, 153102 (2011).

96. Tomiyasu, K., Sato, T & Orimo, S., Estimation of bonding nature using diamagnetic susceptibility. *Chem. Commun.* **51**, 8691 – 8694 (2015).
